# Species-Specific Duplication and Adaptive Evolution of a Candidate Sex Pheromone Receptor Gene in Weather Loach

**DOI:** 10.3390/genes12121845

**Published:** 2021-11-23

**Authors:** Lei Zhong, Weimin Wang, Xiaojuan Cao

**Affiliations:** 1 Fisheries Research Institute, Wuhan Academy of Agricultural Sciences, Wuhan 430207, China; leiclock@163.com; 2 College of Fisheries, Key Lab of Agricultural Animal Genetics, Breeding and Reproduction of Ministry of Education/Key Lab of Freshwater Animal Breeding of Ministry of Agriculture and Rural Affairs, Huazhong Agricultural University, Wuhan 430070, China; wangwm@mail.hzau.edu.cn

**Keywords:** pheromone receptor, olfaction, gene duplication, loach, differential expression, adaptive evolution

## Abstract

The release and sensation of sex pheromone play a role in the reproductive success of vertebrates including fish. Previous studies have shown that the weather loach *Misgurnus anguillicaudatus* perceives sex pheromones by olfaction to stimulate courtship behavior. It was speculated that weather loaches use smell to recognize intraspecific mates. However, the identification of loach pheromone receptor has not been reported. By comparative transcriptomic approach, we found that the olfactory receptor gene *or114-1* was male-biasedly expressed in the olfactory epithelium of *M*. *anguillicaudatus*, *M*. *bipartitus* and the closely related species *Paramisgurnus dabryanus*. This sex-biased expression pattern implicated that *or114-1* presumably encoded a sex pheromone receptor in loaches. *M*. *bipartitus* and *P*. *dabryanus*, like zebrafish, possess one *or114-1* only. However, in *M. anguillicaudatus*, *or114-1* has two members: *Ma_or114-1a* and *Ma_or114-1b*. *Ma_or114-1a*, not *Ma_or114-1b*, showed sex-differential expression in olfactory epithelium. *Ma_or114-1b* has base insertions that delayed the stop codon, causing the protein sequence length to be extended by 8 amino acids. *Ma_or114-1a* was subject to positive selection resulting in adaptive amino acid substitutions, which indicated that its ligand binding specificity has probably changed. This adaptive evolution might be driven by the combined effects of sexual selection and reinforcement of premating isolation between the sympatric loach species.

## 1. Introduction

The sensory system and its signal source play an important role in the pre-mating reproductive isolation between species [1,2]. Similar to the visual system and its signals, the chemical sensory system (including smell and taste) and chemical signals also participate in the evolution of genetic communication barriers [1,3]. Pheromone is a chemical signal, a molecule or a fixed combination of molecules, released by the organism to stimulate the instinctive response of other individuals within the same species, and it is widely present in various groups in the animal kingdom [4]. Pheromone is sensed by smell mainly by binding to olfactory receptors [5,6]. As a type of pheromone, sex pheromone has the function of attracting the opposite sex in the same species, promoting reproductive synchronization and inducing reproductive behavior [7,8]. In the past 10 years, sex pheromones have been identified in many species, including fish, with their extremely diverse chemical properties. For example, they are mostly long-chain hydrocarbons in insects and include steroids and peptides in vertebrates [9,10]. Correspondingly, receptors of sex pheromones have evolved very differently in different animal groups [7].

The sex pheromone-olfactory system is highly species-specified [4]. Pheromone signals can achieve specificity in two ways: using a single unique molecule or a specific combination of multiple molecules [7]. If the genes related to the synthesis of sex pheromone mutate, it may lead to the production of new molecular structures or new combinations of multiple molecules, which will lead to changes in their specificity. On the other hand, variations in the amino acid sequence of sex pheromone receptors will cause changes in the binding specificity of sex pheromone molecules [7]. When the sex pheromone to be sensed is a combination of multiple molecules, what is needed is a combination of multiple sex pheromone receptors [11]. Therefore, the mutation of a single sex pheromone receptor can also bring new specificity of perceiving sex pheromones at the combinatorial level.

In fish, the sex pheromone-olfactory system is related to the successful conduction of the reproduction process, which has been confirmed by the research on the sex pheromone-olfactory system of goldfish and others [12,13,14]. The identified sex pheromones in fish mainly include sex steroids, prostaglandins and bile acids [12]. The identification of fish sex pheromone receptors lags behind that of mammals, while the sex pheromone receptors identified in vertebrates are from olfactory receptors [10]. Olfactory receptors in vertebrates mainly include OR (odorant receptor), TAAR (trace amine-associated receptor), V1R (vomeronasal type 1 receptor) and V2R (vomeronasal type 2 receptor) families, belonging to the 7-transmembrane G protein-coupled receptor super family, with ligand binding sites in the transmembrane domain [15,16]. There are more than 300 members of these four families in zebrafish [17]. OR, TAAR, V1R, and V2R families all have members that assume the function of pheromone receptors in vertebrates [10,18]. Among them, the role of OR114-1 and V1R2 (ORA1) as sex pheromone receptors has been functionally verified in zebrafish [18,19,20].

Through whole-genome sequencing and olfactory epithelial transcriptome sequencing, olfactory receptors can be efficiently identified. In addition to whole-genome sequencing, fishes of which the olfactory epithelial transcriptomes have been sequenced include: *Danio rerio* [21,22], *Carassius auratus* [23], *Senegalese sole* [24], *Anguilla anguilla* [17], *Oncorhynchus keta* [25], *Coilia nasus* [26], *Megalobrama amblycephala* [27], which suggests that transcriptome sequencing is a feasible method for olfactory receptor identification.

Weather loaches (fishes in the genus *Misgurnus* and *Paramisgurnus*), which are of important aquacultural and medicinal values in East Asia, have a well-developed sense of smell due to their benthic living habits and accompanying visual degradation. Studies have shown that weather loaches perceive sex pheromones by olfaction to stimulate courtship behavior [28,29], and it is speculated that they use smell for species-specific mate recognition. Therefore, weather loaches have the potential to be models for the study of sex pheromone and its perception system. However, the direct identification and functional verification of loach pheromone receptors have not been previously reported.

Males have a perception different from that of females to the same sex pheromone. To arouse different perception of one ligand in different sexes, there are mainly two ways: the expression of the receptors is different in different sexes or, the higher brain circuitry is sexually dimorphic. The former is more direct. Therefore, although a sex pheromone receptor is not necessarily sex-differentially expressed, we could expect that a part of sex pheromone receptors might have a sex-differential expression pattern. On the other hand, if an olfactory receptor has a sex-differential expressed pattern in olfactory epithelium, it is highly probable that the olfactory receptor plays a role of sex pheromone receptor.

In this study, by comparative transcriptomic approach, we tried to find candidate sex pheromone receptors in the weather loaches through the identification of male/female differentially expressed olfactory receptors, and to investigate their molecular evolution.

## 2. Materials and Methods

### 2.1. Sample Collection

The sample collection and following experiments were conducted in accordance with the national legislation of China and approved by the Ethics Committee of Huazhong Agricultural University. We collected adult samples of *M*. *anguillicaudatus*, *M*. *bipartitus* and *P*. *dabryanus* in Yueyang, Zhoushan, Daqing and Honghu of China (Table 1). The taxonomic classification of the samples relied on the morphological characteristics given by Chen [30]. The sex identification of weather loach was based on the morphological sexual dimorphism: the males have a small bony plate on each pectoral fin that the females don’t have [31]. Because there are natural diploid and tetraploid populations in *Misgurnus anguillicaudatus* [32,33], we employed a flow cytometry (CyFlow Space, Sysmex Partec, Germany) to detect the DNA content of erythrocytes to determine the ploidy status of each sample. The peripheral blood of each loach was collected from the tail vein using a syringe. Then the blood cells were suspended and stained in 1 mL staining buffer (Cystain DNA 1 step staining solution, Sysmex Partec, Germany) for 1 min. Erythrocytes of *Paramisgurnus dabryanus* have a relative DNA content of diploid and their corresponding fluorescence intensity was used as control. The diploid individuals were leaved for subsequent experiments.

### 2.2. Transcriptome Sequencing and Analyses

#### 2.2.1. Tissue Sampling and RNA Extraction

One pair of male and female mature individuals from each species (*M*. *anguillicaudatus* (B1, B2), *M*. *bipartitus* (C1, C2) and *P*. *dabryanus* (D1, D5)) was sampled during the mating period. After anesthesia, the olfactory epithelium of each individual was dissected under a microscope and then respectively used for total RNA extraction. The total RNA was extracted using TRIzol reagent (Invitrogen, USA). The gDNA eraser (TaKaRa, China) was used to remove the genomic DNA in the process of RNA isolation.

#### 2.2.2. Library Construction and Sequencing

Library preparation and sequencing were carried out by BGI (Shenzhen, China). Briefly, after mRNA isolation of the total RNA, libraries for transcriptome sequencing were constructed using the NEBNext Ultra RNA Library Prep Kit for Illumina. Agilent 2100 Bioanaylzer and ABI StepOnePlus Real-Time PCR System were employed in the qualification and quantification of the libraries. One sequencing library was constructed for each individual. Therefore, there are a total of 6 libraries. Using the Illumina HiSeq 4000 platform (Illumina, San Diego, CA, USA), at least 5Gb data (150 bp paired-end reads) was produced for each library. The derived reads were deposited into the Sequence Read Archive of the National Center for Biotechnology Information (NCBI) (Accession No. SRR14800568-14800573).

#### 2.2.3. Bioinformatic Analyses

##### Sequence Assembly and Annotation

After filtering the data of the next-generation sequencing, the Trinity program was employed for *De novo* assembly [34], and then the Tgicl program was adopted to remove the redundancy to obtain non-redundant transcript sequences [35]. The reads of the three species *M*. *anguillicaudatus*, *M*. *bipartitus* and *P*. *dabryanus* were assembled separately. In this way, the transcript sequence sets of each of the three species could be obtained. The functions of the transcripts were annotated using NT, NR, SwissProt, COG, KEGG and GO databases. The olfactory receptor genes were extracted according to the functional annotation.

##### DEG Detection

First, the Bowtie 2 program was employed to calculate the expression level of the 6 samples, and then the PossionDis method [36] was adopted to detect differentially expressed genes (DEG) between male and female samples of the same species. The intersection of olfactory receptor genes and differentially expressed genes were inferred as candidate sex pheromone receptor genes for subsequent analyses.

##### Ortholog Identification

The CDSs of transcripts were predicted based on the alignments of the functional annotation. Then BLAST and phylogenetic analyses were adopted to determine the orthologous relationships of the sequences. According to the candidate sex pheromone receptor gene sets and the orthology, the orthologous candidate sex pheromone receptor genes of the three species could be obtained. The phylogenetic analyses were performed by the maximum likelihood method (ML) under the general time-reversible (GTR) model in the MEGA7 software [37]. The statistical confidence of each node was assessed with 500 bootstrap replicates. The phylogenetic analyses were also performed by the Bayesian method under the GTR model in the MrBayes 3.1.2 program [38]. Two independent Bayesian analyses were run simultaneously for 10 million generations each sampling every 100th generation. A burn-in of 25,000 trees was removed. The statistical confidence in the nodes of the Bayesian tree was assessed by posterior probabilities.

### 2.3. Genomic PCR, Sequencing and Related Analyses

Genomic DNA was extracted by a high-salt method from the fin rays of each individual loach preserved previously in ethanol (a part of samples in Table 1) and then used as a polymerase chain reaction (PCR) template. Based on the 5’UTR and 3’UTR sequences of the transcripts (Supplementary Materials Additional file 1), primers conserved among the three species were designed. The *or114-1* genes were amplified by PCR for each of the species. For high fidelity, PrimeSTAR Max DNA polymerase (TaKaRa, Dalian, China) was adopted in the amplification reaction.

The reaction system was 50 μL, including 10 pmol per primer, 200 ng genomic DNA and 25 μL the PrimeSTAR Max premix.

PCR program: 95 °C for 5 min, followed by 30 consecutive cycles of the following process: 94 °C for 45 s, 55 °C for 45 s and 72 °C for 1 min.

Primer sequences:

Forward: GGAGAGAGATTTTGTAGATGCTGC;

Reverse: GACAATCACACACAAAAGAGAATG

Each PCR product was purified and sequenced. When double peaks or multiple peaks appeared on the sequencing peak map, the purified PCR product was cloned using the Mighty TA-cloning Reagent Set for PrimeSTAR® kit (TaKaRa, Dalian, China) and *E. coli* DH5α Competent Cells (TaKaRa, Dalian, China). At least 6 positive clones were sequenced from each individual of *M*. *bipartitus* and *P*. *dabryanus* on an ABI 3730 capillary sequencer (Applied Biosystems, Foster City, USA). At least 20 positive clones were sequenced for each individual of *M*. *anguillicaudatus* to ensure that each allele has two monoclonal representatives due to *M*. *anguillicaudatus* having two *or114-1* paralogs inferred from the transcriptomic data. The derived representative gene sequences were deposited to GenBank (Accession No. MZ065164-MZ065167; for allele sequences, see Supplementary Materials Additional file 2).

The phylogenetic analyses of the obtained sequences were conducted by the ML method under the GTR model in the MEGA7 software. The statistical confidence of each node was assessed with 500 bootstrap replicates. The phylogenetic analyses were also performed by the Bayesian method under the GTR model in the MrBayes 3.1.2 program. Two independent Bayesian analyses were run simultaneously for 10 million generations each sampling every 100th generation. A burn-in of 25,000 trees was removed. The statistical confidence in the nodes of the Bayesian tree was assessed by posterior probabilities.

### 2.4. Quantitative RT-PCR Assays

First, three pairs of mature male and female individuals from each of the three species were obtained as biological replicates, and the olfactory epithelium of each individual was sampled (*Misgurnus anguillicaudatus*, female: B1, YYM6, YYM8, male: YYM2, YYM3, YYM4; *M. bipartitus*, female: C1, DQB52, DQB53; male: C2, DQB54, DQB56; *Paramisgurnus dabryanus*, female: D7, YYD5, YYD6; male: YYD1, YYD2, YYD3). Total RNA was extracted from each sample.

Then, the RevertAid First Strand cDNA Synthesis Kit (Thermo Scientific, Vilnius, Lithuania) was used to reverse transcribe mRNA into cDNA.

Next, The Bio-Rad CFX Connect Real-Time PCR Detection System was employed for quantitative RT-PCR (qRT-PCR) analysis. The reagent used was the Power SYBR Green PCR Master Mix (Applied Biosystems). The analyses were carried out separately for each of the three species. Three technical replicates were prepared for each sample. β-actin was used as the internal reference gene and its primers were designed based on conservative sequences of the three species. Primers that specifically amplify *Ma_or114-1a* and *Ma_or114-1b* were designed based on the unique sequences, and were used in qRT-PCR after verification by PCR sequencing. According to the transcript sequences, the primers were designed to amplify *Mb_or114-1* (*M*. *bipartitus*) and *Pd_or114-1* (*P*. *dabryanus*) in qRT-PCR. The primer sequences used are shown in Table 2.

The PCR program is described in Table 1. A melting curve analysis was performed to ensure unique amplification for each primer pair. The relative expression levels of male and female samples of a certain species were quantified by the comparative C_T_ (2^−ΔΔCT^) method [39].

Different individuals of the same species are divided into two groups according to males and females (three biological replicates in each group). Based on the relative quantification of the expression of the same gene in samples of the same species, a two-sample F test was used to evaluate the homogeneity of variance, and then a two-sample t test was conducted to examine whether the gene expression level of male loach was significantly up-regulated relative to that of female loach.

### 2.5. Analysis of Adaptive Evolution

First, based on the *or114-1* sequences of the three loach species, we tested whether the evolution rate of the target branch of the gene tree was significantly different to and faster than that of other branches, that is, whether the target branch’s ω (ω = dN/dS, the ratio of the non-synonymous substitution rate to the synonymous substitution rate) was different to and significantly greater than that of other branches. The codeML program in the PAML package was adopted to perform statistical tests based on the branch model [40]. The input tree is (((*Ma_or114-1a*, *Ma_or114-1b*), *Mb_or114-1*), *Pd_or114-1*). Likelihood values were calculated under one-ratio model and two-ratio model. Under the two-ratio model, the target branch was set as the foreground branch and other branches as the background branch, and the respective ω of the foreground branch and the background branch were calculated. The likelihood ratio test was first performed on the likelihood values under the two-ratio model and the one-ratio model to infer whether the ω of the target branch was significantly different to and significantly greater than that of other branches. Next, the likelihood ratio test was performed on the likelihood values calculated under the two-ratio model and the two-ratio model (limited ω1 = 1) to infer whether the ω of the target branch is significantly greater than 1.

Because positive selection may only occur at a few sites of a specific branch instead of all sites, the next step was to use the codeML program to perform a positive selection test based on the branch-site model [40]. The target branch was set as the foreground branch, and the other branches were set as the background branch. The likelihood ratio test was performed on the likelihood values under Model A and Model A (limited ω2 = 1). At the same time, the BEB method attached to Model A can calculate the posterior probabilities of different types of sites and identify sites subject to positive selection.

The online program RELAX was adopted to test whether the change in the evolution rate of the target branch relative to the reference branch was due to the relaxation of purifying selection or the increase of positive selection [41]. aBSREL [42] and BUSTED [43], which are alternative selection testing methods based on the branch-site model, were also used to run tests for adaptive evolution, and both tests can be performed online. The website of RELAX, aBSREL, BUSTED online programs is: http://www.datamonkey.org (accessed on 8 April 2018).

Transmembrane regions of Ma_OR114-1 was predicted by the TMpred server (https://embnet.vital-it.ch/software/TMPRED_form.html accessed on 25 March 2018) [44].

## 3. Results and Discussion

### 3.1. Identification of a Male-Biasedly Expressed or Gene

In the sex-differentially expressed gene sets preliminarily identified by the bioinformatic analysis on the transcriptomic data of olfactory epithelium, one olfactory receptor gene was found showing male-biased expression in all the three loach species (*M*. *anguillicaudatus*, *M*. *bipartitus* and *P*. *dabryanus*) (Supplementary Materials Additional file 1, Table S1), hence it was presumably a candidate gene encoding sex pheromone receptor. Intriguingly, after checking the gene annotations, we found that this gene was orthologous to zebrafish *or114-1* which has been functionally verified as a sex pheromone receptor [18]. Zebrafish *or114-1* belongs to the A subfamily of *OR* family [45]. In zebrafish, the male mating behavior is stimulated by *or114-1* mediated perception of prostaglandin F2α and its derivatives [18]. Since *or114-1* likely expressed sex-differentially in the olfactory epithelium of the three loach species which belong to the order Cypriniformes but in a basal branch different from zebrafish, this observation together with the previous study raised the possibility that *or114-1* acts as a sex pheromone receptor at least in Cypriniformes.

### 3.2. Species-Specific Duplication of or114-1

**Figure 1:**
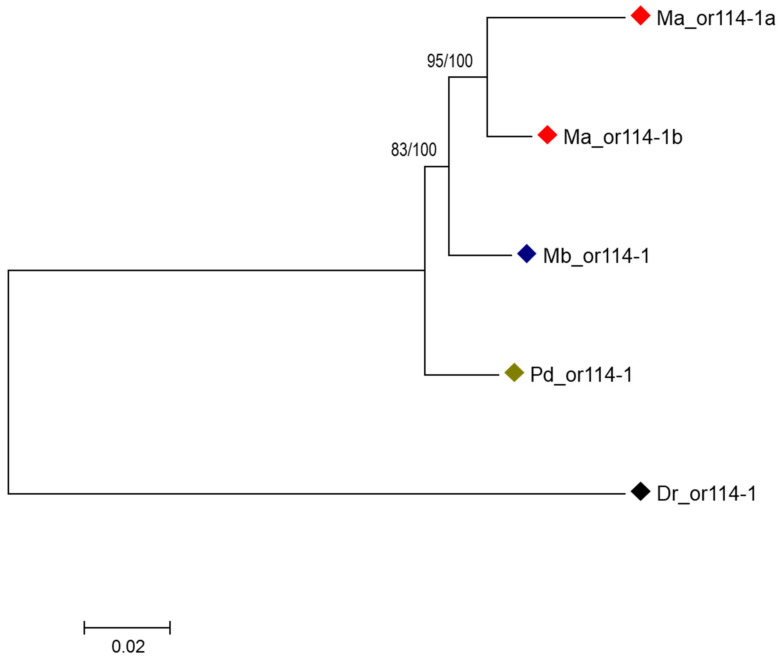
Phylogenetic tree of *or114-1* of the three loach species and zebrafish. ML tree is shown and the general topologies of Bayesian tree is as the same as that of ML tree. Two types of statistical support values for nodes are provided: the former number is the percentage of support calculated with 500 bootstrap replicates in the ML tree reconstruction and the second number is posterior probabilities in the Bayesian reconstruction. Ma, *Misgurnus anguillicaudatus*; Mb, *M. bipartitus*; Pd, *Paramisgurnus dabryanus*; Dr, *Danio rerio*. GenBank Accession number of *Dr_or114-1*: NM_001128582.

In the searching for the *or114-1* sequences in the assembled transcripts, we found that in *M. anguillicaudatus*, there were two distinct *or114-1* haplotypes (named *Ma_or114-1a* and *Ma_or114-1b*) with considerable differences in sequence (more than 40 pairwise differences in 936 positions in the alignment), while *M. bipartitus* and *P. dabryanus*, and also *D*. *rerio*, each have only one *or114-1*. The CDSs of *or114-1a* of *M. anguillicaudatus* and *or114-1* of *M. bipartitus*, *P. dabryanus* and *D. rerio* are all 942 bp (314 aa) in length. *Ma_or114-1b* has insertions within the 3’end of the CDS, resulting in the stop codon being shifted and the CDS length being extended to 966bp (322aa). *Ma_or114-1a* showed male-biased expression in *M. anguillicaudatus* as *or114-1* did in *M. bipartitus* and *P. dabryanus*, whereas there was no significant difference between male and female expression of *Ma_or114-1b* (Table S1). Since there is only one *or114-1* in zebrafish, and also in *M. bipartitus* and *P. dabryanus*, it could be inferred that the two *or114-1* in *M. anguillicaudatus* were paralogs produced by lineage-specific gene duplication. Phylogenetic tree reconstruction was performed using the ML method based on the alignment of *or114-1* sequences of the three loach species and zebrafish. In the resulting gene tree, the two *or114-1* of *M. anguillicaudatus* first clustered with each other, then together clustered with *or114-1* of *M. bipartitus* (Figure 1). This tree topology suggests that the gene duplication event which produced *Ma_or114-1a* and *Ma_or114-1b* in the genome of *M. anguillicaudatus* occurred in the branch leading to *M. anguillicaudatus* after the divergence of the ancestors of *M. anguillicaudatus* and *M. bipartitus*, that is, this gene duplication is species-specific to *M. anguillicaudatus*.

**Figure 2:**
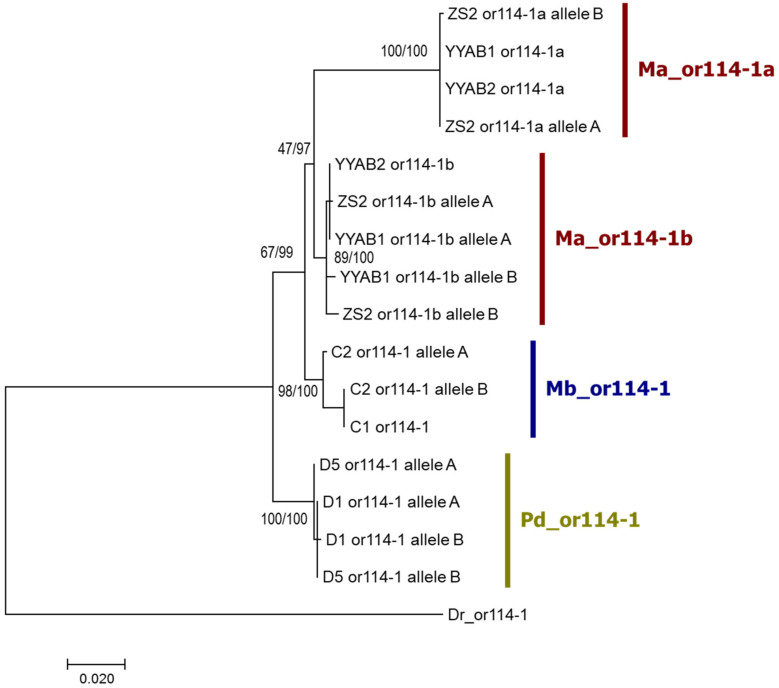
Phylogenetic tree of *or114-1* alleles of the three loach species. *or114-1* of zebrafish was used as an outgroup. ML tree is shown and the general topologies of Bayesian tree is as the same as that of ML tree. Two types of statistical support values for nodes are provided: the former number is the percentage of support calculated with 500 bootstrap replicates in the ML tree reconstruction and the second number is the posterior probabilities in the Bayesian reconstruction. Abbreviations of species names are as in Figure 1. Individual numbers are as in Table 1.

In order to determine whether the two *or114-1* (*Ma_or114-1a* and *Ma_or114-1b*) of the weather loach are indeed paralogs rather than alleles, we surveyed the *or114-1* sequences of more individuals. If *Ma_or114-1a* and *Ma_or114-1b* are paralogs in the weather loach genome, then it is expected that *Ma_or114-1a* and *Ma_or114-1b* exist in every individual of the loach, and possibly each of the two paralogs has two different alleles in some individuals. If *Ma_or114-1a* and *Ma_or114-1b* are actually two alleles in one locus instead, then only one of the two *or114-1* types would appear in some individuals because this locus has a probability to be homozygous. We conducted genomic PCR using conservative primers and subsequent cloning and sequencing to obtain the *or114-1* haplotype sequences of several loach individuals (three of *M. anguillicaudatus*, two of *M. bipartitus*, and two of *P. dabryanus*), and then underwent phylogenetic analysis (using zebrafish *or114-1* as an outgroup). The result showed that different individuals of *M. anguillicaudatus* all possessed the two *or114-1* types which were initially identified as paralogs; *Ma_or114-1a* and *Ma_or114-1b* each have 1 to 2 alleles in each individual; individuals of *M. bipartitus* and *P. dabryanus*, the same as zebrafish, each only possessed one *or114-1* sequence or two *or114-1* sequences which were slightly different from each other (less than 10 pairwise differences in 936 positions in the alignment) and thus more likely alleles rather than paralogs (Figure 2; Supplementary Materials Additional file 2). Therefore, it was proven that *Ma_or114-1a* and *Ma_or114-1b* of *M. anguillicaudatus* are not alleles, but true paralogs.

Additionally, taking advantage of the ongoing loach genome sequencing project of the weather loach, we performed BLAST searches with the assembled sequences obtained by genome sequencing and found that *Ma_or114-1a* and *Ma_or114-1b* were in the same chromosome but not in the same contig. We compared the 5kb flanking sequences both upstream and downstream of *or114-1* in the two contigs and found there to be no obvious similarity between the flanking regions of the contigs (Supplementary Materials Additional file 3). This result indicated that the two *or114-1* were located at different loci of the loach genome, thus further supporting that *Ma_or114-1a* and *Ma_or114-1b* are paralogs produced by gene duplication. Meanwhile, whether the duplication of *or114-1* in the loach took place by tandem duplication, segmental duplication or retrotransposition is still an open question.

### 3.3. qRT-PCR Verification of the Sex-Differential Expression

**Figure 3:**
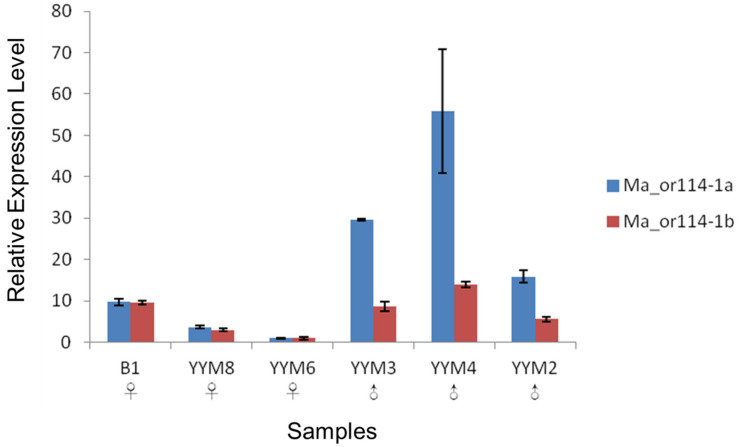
The relative expression levels of *Ma_or114-1a* and *Ma_or114-1b* in different male and female individuals of *Misgurnus anguillicaudautus*, based on the relative quantitative results of qRT-PCR (2^–^*^∆∆^*^CT^). Three technical replicates for each individual and each gene, and the error bar is the standard deviation of the three technical replicates. The sample numbers are as shown in Table 1. When comparing expression difference of *Ma_or114-1a* between the male group and the female group, F = 0.04824, *P* = 0.09203 > 0.05; *t* = −2.41623, *P* = 0.03653 < 0.05 (equal variance assumed); significant. When comparing expression difference of *Ma_or114-1b* between the male group and the female group, F = 1.123, *P* = 0.94206 > 0.05; *t* = −1.37163, *P* = 0.12104 > 0.05 (equal variance assumed), not significant.

We added biological replicates for each species to perform quantitative expression analyses of *or114-1* by qRT-PCR to verify the preliminary conclusion of male-biased expression drawn from the bioinformatic analysis on RNA-seq data. Based on the qRT-PCR derived data, statistical tests were conducted to identify the significance of the expression difference between males and females. To the expression of *Ma_or114-1a*, the homogeneity of variance of the male group and the female group was not refused according to the result of F-test (F = 0.04824, *P* = 0.09203 > 0.05), and the *t*-test under the assumption of variance homogeneity produced a significant result (*t* = −2.41623, *P* = 0.03653 < 0.05), which indicated that the expression of *Ma_or114-1a* in males was significantly higher than that in females (Figure 3). The same method was used to analyze the expression of *Ma_or114-1b*, and the result showed that there was no significant difference between males and females (Figure 3), suggesting that *Ma_or114-1b* might have undergone neo- or subfunctionalization and no longer assumed the previous role of sex pheromone receptor. The same analysis was also performed on the expression of *or114-1* in *M. bipartitus* and *P. dabryanus*. The results showed that in these two species the expression levels in males were all higher than those in females of the same species despite the difference not being statistically supported (*P* > 0.05, *t*-test) (Figures S1 and S2). The insignificant *t*-test results were possibly caused by that the expression levels of *or114-1* in olfactory epithelium differed greatly between male individuals, which resulted in large variance, and the number of biological replicates was relatively limited.

### 3.4. Adaptive Evolution of or114-1 in Weather Loach

In the reconstructed gene tree of *or114-1*, including orthologs of *M. anguillicaudatus*, *M. bipartitus*, *P. dabryanus* and *D. rerio* (as the outgroup), the *or114-1*s of *M. anguillicaudatus*, namely *Ma_or114-1a* and *Ma_or114-1b*, clustered together (Figure 1), suggesting that they were products of a gene duplication event that occurred in *M. anguillicaudatus* after the divergence of *M. anguillicaudatus* and *M. bipartitus*. Meanwhile, the branch of *Ma_or114-1a* was significantly longer than that of *Ma_or114-1b*, and also longer than the branches of *or114-1* of *M. bipartitus* and *P. dabryanus* (Figure 1), suggesting that the evolution rate of *Ma_or114-1a* has accelerated. This acceleration of evolution rate could be caused by either positive selection or relaxed purifying selection. In order to test the prediction of adaptive evolution of *Ma_or114-1a* under positive selection, we conducted a series of tests based on the *or114-1* sequences of *M. anguillicaudatus*, *M. bipartitus* and *P. dabryanus* using multiple tools.

We first employed the codeML program to perform the statistical test based on the branch model [40]. In the first step, we test whether the evolution rate of the branch *Ma_or114-1a* was significantly faster than that of other branches, that is, whether the ω of the branch *Ma_or114-1a* was significantly larger than that of other branches. Under the two-ratio model, the ω (1.461) of the *Ma_or114-1a* branch set as the foreground branch was larger than the ω (0.136) of the other branches as the background branch. The likelihood ratio test was performed on the likelihood values under the two-ratio model and the one-ratio model, and the result was significant (*P* < 0.001) (Table 3), indicating that the evolution rate of *Ma_or114-1a* branch was significantly faster than other branches. In the next step, we test whether the branch *Ma_or114-1a* was subject to positive selection under the branch model, that is, whether the ω of this branch was significantly greater than 1. The likelihood ratio test was performed on the likelihood values under the two-ratio model and the two-ratio model (limited ω1 = 1), and the statistical result was not significant (*P* > 0.05) (Table 3). Therefore, the test based on the branch model was ineffective to tell whether the branch of *Ma_or114-1a* was subject to positive selection or relaxed selective constraints.

The RELAX test can be used to determine whether the acceleration of evolution rate of the target branch relative to the reference branch for a certain gene tree is due to the relaxation of purifying selection pressure or the increase of positive selection pressure [41]. Based on the *or114-1* sequences of *M. anguillicaudatus*, *M. bipartitus* and *P. dabryanus*, we set *Ma_or114-1a* as the target branch and performed RELAX test. The result of the test for selection intensification was significant (K = 12.30, *p* < 0.001) (Table S2), indicating that the selection pressure on the *Ma_or114-1a* branch has increased rather than relaxed, namely, the accelerated evolution of *Ma _or114-1a* was caused by positive selection rather than relaxed purifying selection.

**Figure 4:**
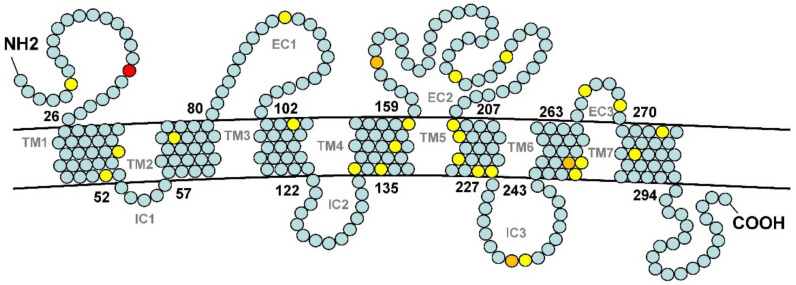
The predicted protein structure of Ma_OR114-1a of *M. anguillicaudatus*. The detected amino acid sites subject to positive selection are indicated in red (posterior probability < 0.01), orange (*P* < 0.05) and yellow (*P* < 0.1). It is worth noting that point mutations in the transmembrane domains TM3, TM5, and TM6 can change the ligand binding specificity of the olfactory receptor. IC: inside the membrane; EC: outside the membrane.

The branching model is not highly effective in testing positive selection due to the fact that positive selection may only occur at a few sites rather than all sites in a gene. Next, we performed a positive selection test based on the branch-site model in the codeML program [40]. The *Ma_or114-1a* branch was set as the foreground branch, and the other branches were set as the background branch. The likelihood ratio test was performed on the likelihood values under Model A and Model A (limited ω2 = 1), and the statistical result was significant (*P* < 0.05) (Table 4). This result supported that *Ma_or114-1a* has been positively selected at some sites after diverging from *Ma_or114-1b*. At the same time, as the BEB method attached to Model A can be used to calculate the posterior probability of different site types, 28 sites subject to positive selection were identified (Table 4). Compared with the predicted transmembrane domain protein model based on the amino acid sequence, it was found that positive selection sites were distributed in the 7 transmembrane domains, 3 extramembrane loops, the third inner loop and the N-terminal out of the membrane (Figure 4). Among them, the TM3, TM5, and TM6 transmembrane domains, which combine to form a pocket structure, have 1, 5, and 3 positively selected amino acid sites, respectively. Previous studies have confirmed that point mutations on TM3, TM5, and TM6 can change the ligand binding specificity of olfactory receptors [46]. The amino acid substitutions in these three domains on *Ma_or114-1a* were expected to change the binding profile of ligands. These amino acid substitutions that led to changes in ligand binding specificity were subject to positive selection, which means that these amino acid substitutions brought adaptive evolution.

At the same time, we performed aBSREL and BUSTED tests based on the branch-site model to further verify whether *Ma_or114-1* was subject to positive selection. Both tests yielded statistically significant results (*P* < 0.05, Tables S3 and S4), which further supported that at least one amino acid site of *Ma_or114-1a* has been positively selected during evolution.

Genes related to reproduction often have a faster evolutionary rate due to sexual selection [3]. If as inferred, OR114-1 has the function of sex pheromone receptor and is closely related to mate recognition, one plausible explanation for the force of adaptive evolution of *Ma_or114-1a* would be sexual selection. The ancestral OR114-1 might be pluripotency and have other basic functions besides the sex pheromone receptor function (e.g., functions for perceiving the odors of food); hence it expressed both in males and females, and might be subject to strict evolutionary constraints in *M. anguillicaudatus* before the gene duplication and in the related species. According to the evolutionary model of gene duplication [47], after the duplication of *or114-1* in *M. anguillicaudatus*, the two copies then might undergo possible functional differentiation. *Ma_or114-1b* probably inherited some other basic functions (subfuntionalization), while *Ma_or114-1a* more specifically assumed the role of sex pheromone receptor. As a result, the evolutionary constraints imposed on *Ma_or114-1a* were relaxed, namely purifying selection is weakened, while the driving force of sexual selection would be prominent, hence the amino acid sequence was subject to positive selection and adaptive amino acid substitution occurred.

Since *M. anguillicaudatus* and *P. dabryanus* live in the same habitats and the breeding seasons overlap, there might be a process of reinforcement between the two species [48,49,50]. This process strengthens the pre-mating reproductive isolation through the differentiation of mating-related traits of the two species [51,52]. Due to the similar morphology and benthic life of *M. anguillicaudatus* and *P. dabryanus*, the role of visual signals in mating behavior is presumed to be relatively weak, whereas the role of olfactory signals is believed to be prominent, hence the sex pheromone-receptor system probably plays a key role in species recognition during mating. The selection for the divergence of the sex pheromone-receptor systems of the two species in reinforcement likely have promoted the adaptive evolution of *Ma_or114-1a*.

## 4. Conclusions

Through trans-species comparative transcriptomic analysis and qRT-PCR assay of three closely related loach species, we found a male-biased expression pattern of the olfactory receptor gene *or114-1* shared by the three species. Hence it was inferred that OR114-1 might be a sex pheromone receptor in the loach species, which was in concordance with the OR114-1 function in zebrafish that was revealed in the previous study. As with zebrafish, *M. bipartitus* and *P. dabryanus* both possessed one *or114-1*; whereas in *M. anguillicaudatus*, *or114-1* has two members: *Ma_or114-1a* and *Ma_or114-1b*. Among the two *or114-1* members of *M. anguillicaudatus*, the one with sex-differential expression is *Ma_or114-1a*. These two members have undergone specific changes: *Ma_or114-1b* was delayed of the stop codon due to insertions, and the protein sequence length was extended by 8 amino acids; *Ma_or114-1a* underwent positive selection, and the adaptive amino acid substitution occurred. The ligand binding specificity of the duplicates relative to the ancestral receptor before the duplication probably has changed considering the tremendous sequence change, which implicates the recognition profile of the sex pheromone of the receptors in change. This adaptive evolution might be driven by the co-effect of sexual selection and reinforcement.

## Figures and Tables

**Table 1:** Sample information.

Species	Sample Number	Sex	Location
*Misgurnus anguillicaudatus*	B1	Female	Yueyang
	B2	Male	Yueyang
	YYAB1	Female	Yueyang
	YYAB2	Male	Yueyang
	YYM2	Male	Yueyang
	YYM3	Male	Yueyang
	YYM4	Male	Yueyang
	YYM6	Female	Yueyang
	YYM8	Female	Yueyang
	ZS2	Female	Zhoushan
*Misgurnus bipartitus*	C1	Female	Daqing
	C2	Male	Daqing
	DQB52	Female	Daqing
	DQB53	Female	Daqing
	DQB54	Male	Daqing
	DQB56	Male	Daqing
*Paramisgurnus dabryanus*	D1	Female	Honghu
	D5	Male	Honghu
	D7	Female	Honghu
	YYD1	Male	Yueyang
	YYD2	Male	Yueyang
	YYD3	Male	Yueyang
	YYD5	Female	Yueyang
	YYD6	Female	Yueyang

**Table 2:** Primers for qRT-PCR.

Primer Name	Primer Sequence (5′-3′)	TM (°C)	Product Length (bp)
*β-actin* F	GGGTATGGAGTCTTGCGGTA	58.88	131
*β-actin* R	CAGCAATGCCAGGGTACATG	59.26	
*Ma_or114-1a* F	AAGATCTACGTGTGATATGTACC	59.08	140
*Ma_or114-1a* R	TTAATGCAGATTAACCTTATTAGAAG	58.20	
*Ma_or114-1b* F	GAGATCTACGTGTGCTATGTAC	58.89	150
*Ma_or114-1b* R	GGGAAGACCACTGATGTAGAT	58.78	
*Mb_or114-1* F	GCCATCATTTTCCCCTTGCA	59.10	119
*Mb_or114-1* R	CCCAGCAAACCAACCATTGA	58.95	
*Pd_or114-1* F	TGTGTTTGTGAACACACATCA	58.00	163
*Pd_or114-1* R	CGCCTGAAGGAGAACGAAAT	59.72	

The PCR program used in the experiment is: (1) 95.0 °C for 3:00 min (2) 95.0 °C for 0:10 min (3) 55.0 °C for 0:20 min (4) 72.0 °C for 0:20 min (5) 75.0 °C for 0:05 min (+ Plate Read) (6) GOTO 2, 40 more times (7) Melt Curve 65.0 to 95.0 °C, increment 0.5 °C/s (0:05 min + Plate Read).

**Table 3:** Test result under branch model by codeML.

Model	*log* L	Parameters	LRT
M0 (one-ratio)	−1717.957	ω = 0.376	M0 vs. two-ratio: 2△ι = 19.57, df = 1, *p* < 0.001. two-ratio (ω1 = 1) vs. two-ratio: 2△ι = 0.67, df = 1, *p* > 0.05
two-ratio model	−1708.171	ωbackground = 0.136, ωforeground = 1.461	
two-ratio model (ω1 = 1)	−1708.506	ωbackground = 0.137, ωforeground = 1.000	

**Table 4:** Test result under branch-site model by codeML.

Model	*log* L	LRT	Sites under Positive Selection
Model A	−1705.590	2△ι = 4.04, df = 1, *p* < 0.05	6 L, 20 S **, 41 S, 50 V, 74 Q, 90 K, 105 M, 136 F, 138 T, 148 F, 158 T, 164 I *, 188 I, 193 R, 208 T, 215 F, 219 V, 225 M, 226 S, 234 W *, 235 H, 247 A, 248 M, 249 F *, 265 S, 269 K, 274 I, 281 V
Model A (ω2 = 1)	−1707.570		

NOTE. The amino acid sites with the **, * or no superscript denote the sites with the posterior probability < 0.01, *P* < 0.05 and *P* < 0.1 respectively.

## Data Availability

Publicly available datasets were analyzed in this study. This data can be found here: [SRA Accession No. SRR14800568-14800573; GenBank Accession No. MZ065164-MZ065167].

## Supplementary Materials

The following are available online at https://www.mdpi.com/article/10.3390/genes12121845/s1, Additional file 1: Alignment of representative transcripts of *or114-1* of the three loach species, Additional file 2: Allele sequences of *or114-1* of the three loach species, Additional file 3: Contigs containing or114-1, Figure S1: The relative expression levels of *or114* in different male and female individuals of *Misgurnus bipartitus*, Figure S2: The relative expression levels of *or114-1* in different male and female individuals of *Paramisgurnus dabryanus*, Table S1: Gene differential expression analysis based on RNA-seq data, Table S2: Test result of RELAX, Table S3: Test result of positive selection by aBSREL, Table S4: Test result of positive selection by BUSTED.
