# Supplementary material for: Species-Specific Duplication and Adaptive Evolution of a Candidate Sex Pheromone Receptor Gene in Weather Loach
Source: Genes (Basel). 2021 Nov 23;12(12):1845. doi: 10.3390/genes12121845 (PMC8701048; doi:10.3390/genes12121845)
Supplement: Supplementary file 1 [file genes-12-01845-s001.zip › Supplementary Materials-Additional files.pdf]

Additional File 1. Alignment of representative transcripts of or114-1 of the three loach species

>Ma_or114-1a_TR121279_B
ACTCATTGTCCCAACCTTGTATCAGGGGAGGGGAGCAAGGAATATTTAAGAGAGGCAC
CACTTTGTATTAAATCAGTAGGGGAGAGAGATTTTGTAGATGCTGCTATCGCAGACA-----
---------AAAACTGTTG--
TTTTTTATCTTATGTTAAGAATTGCTTTAGTATTTAACTGATAAAAATATAGATGC-
TTTATTTATTTCAAATCTGCACATGAAATGTAATAACATATTTGATTTGGACAACATTAAT
GCTTAAATTGAACTTTCACCTAAGGAATGGATTATAGGACATTAAACTTTTCTCATAACG
AATTCTACTTGATTGGATTCACGAGTCTAAACGAGTTTCGTCAGTATCTGTTCATCCCAT
TTTTAATCTTGTTTGTGTTCCAATTGTCTGCGAATGGAATTATAATGTTTGTGGTTATAAA
AGAAAGAAAGTTGCATGCTCCCATGTACATTCTGATTGGGTTCATTGCAGCTCTGGGCT
TTATACAACCACTATTTCTTCTTCCAAGAATGTTAGTCAGTTTTCTCTTTGGCAAAAATA
TGATTTATAGAAACGAATGTCTCATTCAAATGTTTTGTATGCATATGGCGGGTTTGTTCC
AGTCAAGTATATTGGTCATGATGGCAGTGGACCGCTTTTTCGCCATCATTTTCCCCTTGC
AGTACCATGACTTTGTAACCTTTAAAACCTCATTAATATTCTGTTTGTTTCTTTTTATCCG
TAACTTGCTTTATGTTACCTCAATGGTTGGTTTGATTGGGCCACTATATTTTTGTAAATCA
AACGAGATTTACCACTGTGTTTGTGAGCATACATCTGTTGTTAATATAGCCTGTGGTGAT
CGTACTAAGAATTACATGGCTGGGATAATCGGTTTCAGTTTAACAACAAGCGACTGTGT
ATTCATTTTCTGTTCTTATGTTGTTATTTTTGTGATTATGTCTCGTTCTCCTTCAGGGGAG
TCGTGGCATAAAGCTATCTATACATGTAGCACACATTTAATGGCCATGTTTGTGGCCTTT
CTCTCTGTTGTGGGTGCATTTGCTGGCTATAGGATTTCTACAATACCTAAAGATCTACGT
GTGATATGTACCTTGGGATATCTTGTTCTTCCGAACTGTTTTAACCCAGTAATCTATGGC
ATGAGAACCAAGGAAATCAGAGTTCAAATTGTTAAATATTTGTCTTCTAATAAGGTT-----
----
AATCTGCATTAACCCCTATGATATGGTTTCATTTGTTGTAGGCTATCTTTGTTTTCTGTTC
CAACTTACTGCGTACATGGATGATGTTTGTAATTAGTCTGTAATTTGTACATACATGTTAA
TAATACATCAACT-
TTTTGTTAGATTGATACAGTAGACGAATTGGTAGAACAGATATGCATCTATTCATTCTCT
TTTGTGTGTGATTGTCATATGCGTACATACCTAAAAGCATCCTGAAATTTTGTTATGACT
GTATATGTAACATTTC---
ATTCTGGTATGATTATAAACATGCCTTTACAAATAAAATGCAACAAAGTTGTGTTGGACT
GTTGTGTT--TCATTCTGTGAAATGTACTGGCTACTCATGTTTACAC------------------------
-T--

>Ma_or114-1b_TR121278_B
ACTCATTGTCCCAACCTTGTATCAGGGGAGGGGAGCAAGGAATATTTAAGAGAGGCAC
CACTTTGTATTAAATCAGTAGGGGAGAGAGATTTTGTAGATGCTGCTATCGCAGACA-----
---------AAAACTGTTG--
TTTTTTATCTTATGTTAAGAATTGCTTTAGTATTTAACTGATAAAAATATAGATGC-
TTTATTTATTTCAAATCTGCACATGAAATGTAATAAAATATTTAATTTGGACAACATTAAT
GCTTAAATTGAACTTTCACCAAAGGAATGGATTATAGGACATTCAACTTTTCTCATAAC
GAATTCTACTTGATTGGATTCACGGATCTAAACGAGTTTCGGCAGTATCTGTTCATCCCA

TTTTTAGTCTTGTTTGTGTTCCAATTGTTTGCGAATGGAATTATAATGTTTGTGGTTATAA
AAGAAAGAAAGTTGCATGCTCCCATGTACATTCTGATTGGGTTCATTGCAGCTCTGGGC
TTTATAGAACCACTATTTCTTCTTCCAAAAATGTTAGTCAGTTTTCTGTTTGGCAGAAAT
GTGATTTATAGAAACGAATGTTTCATTCAAATGTTTTGTCTGCATATGGCCGGTTTATTC
CAGTCAAGTATATTGGTCATGATGGCAGTGGACCGCTTTTTCGCCATCATTTTCCCCTTG
CAGTACCATGACTTTGTAACCTTTAAAACCTCATTAATATTCTGTTTGTTTCTTTTTATCC
GTAACTTGCTTTATGTTACCTCAATGGTTGGTTTGATTGGGCCACTATATTTTTGTAAATC
AAACGAGATTTACCACTGTGTTTGTGAGCATACATCTGTTGTTAATTTAGCCTGTGGTG
ATCTTACTAAGAATTACATGGCTGGGATAATCGGTTTCAGCTTAACATCAAGCGACTGT
GTATTCATTGTCTGTTCTTACATTGTTATTTTTGTGATTATATTTCGTTCTCCTTCAGGGGA
GTCGCGCCAAAAAGCTATCTATACATGTAGCACACATTTAATGGTCTTGGTGGTGGCCT
TTCTCTCTGTTGTGGGTGCATTTGCTGGCTATAGGATTCCTACAATACCTAGAGATCTAC
GTGTGCTATGTACCTTGGGATATCTTATTCTTCCGAACTGTTTTAACCCAGTAATCTATGG
CATGAGAACCAAGGAAATCAGAGTTCAAATTGTTAAATATTTATCTTCTAATAAGGTTA
ATCTACATCAGTGGTCTTCCCTATTTTTTTCATGAGAGCCACACTGATACAGTAGGTAGA
ACCCAAAATGAAGAACACTGATCTACATTAA-CCCTGAGATATGGTTTAATTTGTTG-----
TATCTTTGTTCTCTGTTCCAACTTACTGTGTACATGGATGATGTTTGTAATTAGTCTGTAA
TTTGTACATACATGTTAATAATACATCAACT-
TTTTGTTAGATTGATACAGTAGACGAATTGGTAGAACAGATATGCATCTATTCATTCTCT
TTTGTGTGTGATTGTCATATGCGTACATACCTAAAAGCATCCTGAAATTTTGTTATGACT
GTATATGTAACATTTC---
ATTCTGGTATGATTATAAACATGCCTTTACAAATAAAATGCAACAAAGTTGTGTTGGACT
GTTGTGTT--TCATTCTGTGAAATGTACTGGCTACTCATGTTTACAC--------------------------
-T---

>Mb_or114-1_TR168214_C

AGGGGAGAGAGATTTTGTAGATGCTGCTATTGCAGACATGCTGTAAAAATGACGAACA
GTTG--
TTTTTTATCTTATGTTAAGAATTGCTTTAGTATTTAACTGGTCAAAATATAGAGGC-
TTTATTTATTTCAAATCTGCACATGAAATGTAATAAAATACATT-------
TAACTTTAATGCTTAAATTGAACTTTCACCTAAGGAATGGATTATAGGACATTCAACTTT
TCTCATAACGAATTCTACTTGATTGGATTCACGGATCTAAAAGAGTTTCGGCAGTATCTG
TTCATCCCATTTTTATTCTTGTTTGTGTTCCAATTGTTTGCGAATGGAATTATAATGTTTG
TGATTATAAAAGAAAGAAAGTTGCACGCTCCCATGTACATTCTGATTGGGTTCATTGCA
GCTTTGGGCTTTATAGAACCACTATTTCTTCTTCCAAGGATGTTAGTCAGTTTTCTGTTT
GGCAGAAATATGATTTATAGAAACGAATGTCTCATTCAAATGTTTTGTCTGCATATGGCT
GGTTTGTTCCAGTCAAGTATATTGGTCATGATGGCAGTGGACCGATTTTTTGCCATCATT
TTCCCCTTGCAGTACCATGACTATGTAAACTTTAAAACCTCATTAATATTTTGTTTGTCTC
TTTATATCCGTAACTTGCTTTATGTTGCCTCAATGGTTGGTTTGCTGGGGCCACTATATTT
TTGTAAATCAAACGAGATTTACCACTGTGTTTGTGAGCATACATCTGTTGTTAATTTAGC
CTGTGGTGATCTTACTAAGAATTACATGGCTGGTGTAATCGGTTTCAGCTTAACATCAA
GCGACTGTGTATTCATTGTCTGTTCTTACATTGTTATTTTTGTGATTATATTTCGTTCTCCT
TCAGGTGAGTCACGACAAAAAGCTATCTATACATGTAGCACACATTTAATGGTCTTGGT

GGTGGCCTTTCTCTCTGTTGTGGGTGCATTTGCTGGCTATAGGATTCCTACAATACCTAG
AGATCTACGTGTGCTATGTACCTTGGGATATCTTATTCTTCCGAACTGTTTTAACCCAGT
CATCTATGGCATGAGAACCAAGGAAATCAGAGTTCAAATTGTTAAATATTTGTCTTCTA
ATAAGGTT--
AATCTACATTAA-CCCTGTGATATGGTTTCATTTGTTG-----
TATCTTTGTTCTCTGTTCCAACTTACTGTGTACATGGATGATGTTTGTAATTAGTCTGTAA
TTTGTACATTCATGTTAATAATACATCAACGATTTTGTTAGATTGATACAGTACATGAATT
GGTAGAACAGATATGCATCTATTCATTCTCTTTGTGTGTGATTGTCATATGCATACA----
TAAAAGCAACCTGAAATTTTGTTATGACTGTATATGTAACATTTTTTAATTTGGTATGAT
TATAAACATGCCTTTACATATAAAATGCAACAAAGTTGTGTTGGACTGTTGTGCTTATCA
TTCTGTGAAATGTACTGTCTATTTGTTAAAGGAA-----------------------------
CAGTATGTAGGATTGTGGCCAAAACTGGTATTGCAATCACAAAACTTGTGGCTAAAGCT
GGTACTGCAATCACACAACTGGTGGCCAATACACAAAATGACAACATAAACATCAGTT
GAGG
>Pd_or114-1_TR205479_D

CCACTTTGTATTAAATCAGTAGGGGAGAGAGATTTTGTAGATGCTGCTATTGCAGACAT
GCTGTAAAAACGTCAAACTGTTGTTTTTTTATCTTATGTTAAGAATTGCTTTAGTATTTA
ACTGATAAAAATATAGAGGCTTTTATTTATTTCAACTCTGCGCATGAAATGTATTAAAAT
ATTTAATTTGGACAACATTAATGCTTAAATTGAACTCTCACCCAAGGAATGGATTATAGG
ACATTCAACTTTTCCCATAACGAATTCTACTTGATTGGATTCACGGATTTGAAAGAGTTT
CGGCAGTATCTGTTCATCCCATTTTTAGTCTTGTTTGTTTTCCAATTGTTTGCGAATGGA
ATTATAATGTTTGTGATTATAAAAGAAAGAAAGTTGCATGCTCCCATGTACATTCTGATT
GGGTTCATTGCGGCTCTGGGCTTTATAGAACCAATATTTCTTCTTCCAAGAATGTTAGTC
AGTTTTCTGTTTGGCAGAAATATGATTTATAGAAACGAATGTCTCATTCAAATGTTTTGT
CTGCATATGGCGGGTTTGTTCCAGTCAAGTATATTGGTCGGGATGGCAGTGGACCGATT
TTTTGCCATCATTTTCCCCTTGCAGTACCATGAGTATGTAAACTTTAAAACCTCATTAAT
ATTCTGTTTGTCTCTTTTTATCCGTAACTTGCTTTATGTTGCCTCAATGGTTGGTTTGCTT
GGTCCACTCTATTTTTGTAAATCAAACGAGATTTACCACTGTGTTTGTGAACACACATC
AGTTGTTAATTTAGCCTGTGGTGATCTTACTAAGAATTACGTGGCGGGTGTAATCGGTTT
CAGTTTAACATCAAGCGACTGTGTATTCATTGTCTGTTCTTACATTGTTATTTTTGTGATT
ATATTTCGTTCTCCTTCAGGCGAGTCGCGCCAAAAAGCTATCTATACATGTAGCACACAT
TTAATGGTCTTGGTCGTGGCCTTTCTCTCTGTTGTGGGTGCATTTGCTGGCTATAGGATT
CCTTCAATACCTAGAGATCTACGTGTGCTATGTACCTTGGGATATCTTATTCTTCCGAACT
GTTTTAACCCAGTAATCTATGGCATGAGAACCAAAGAAATCAGAGTTCAAATTGTTAAA
TATTTATCTTCTAATAAGGTT---
-AATCTACATTAA-CCTTGAGATATGGTTTCATTTGTTG-----
TATCTTTGTTTTCTGTTCCAACTTACTGTGTACATGGATGATGTTTGTAATTAGTCTGTAA
TTTGTACATACATGTTAATAATACATCAAC--
TTTTGTTAGATCGCTACAGTACATGAATTGGTAGAACAGATATGCATGTATTCATTCTCTT
TTGTGTGTGATTGTCATATGCATACATACCTATAAGCAACCTGAAATTTTCTTAAGACTG
TATATGTAACATTTA---
ATTCTGGTATGATTATAAACATGCCTTTACAAATAAAATGCACCAAAGTTGTGTTGGACT
GTTGTGTTTATCATTCTGTGAAATGTACTGGCTACTCATGTTTACACTTCATTCATTTTCT

CCAATGACAAACATTTGTCCTATTTGTTGAGTGATCCTATGTTTTTCCTAGTGTATGAAA
ATGTATTCGGTGACTGCATTTGCCTTTTGCACTTATCAATCTTTTTACAATTAAATGACTC
A--------------

Additional File 2. Allele sequences of or114-1 of the three loach species

>YYAB1_or114-1a
ATGGATTATAGGACATTAAACTTTTCTCATAACGAATTCTACTTGATTGGATTCACGAGT
CTAAACGAGTTTCGTCAGTATCTGTTCATCCCATTTTTAATCTTGTTTGTGTTCCAATTGT
CTGCGAATGGAATTATAATGTTTGTGGTTATAAAAGAAAGAAAGTTGCATGCTCCCATG
TACATTCTGATTGGGTTCATTGCAGCTCTGGGCTTTATACAACCACTATTTCTTCTTCCA
AGAATGTTAGTCAGTTTTCTCTTTGGCAAAAATATGATTTATAGAAACGAATGTCTCATT
CAAATGTTTTGTATGCATATGGCGGGTTTGTTCCAGTCAAGTATATTGGTCATGATGGCA
GTGGACCGCTTTTTCGCCATCATTTTCCCCTTGCAGTACCATGACTTTGTAACCTTTAAA
ACCTCATTAATATTCTGTTTGTTTCTTTTTATCCGTAACTTGCTTTATGTTACCTCAATGGT
TGGTTTGATTGGGCCACTATATTTTTGTAAATCAAACGAGATTTACCACTGTGTTTGTGA
GCATACATCTGTTGTTAATATAGCCTGTGGTGATCGTACTAAGAATTACATGGCTGGGAT
AATCGGTTTCAGTTTAACAACAAGCGACTGTGTATTCATTTTCTGTTCTTATGTTGTTAT
TTTTGTGATTATGTCTCGTTCTCCTTCAGGGGAGTCGTGGCATAAAGCTATCTATACATG
TAGCACACATTTAATGGCCATGTTTGTGGCCTTTCTCTCTGTTGTGGGTGCATTTGCTGG
CTATAGGATTTCTACAATACCTAAAGATCTACGTGTGATATGTACCTTGGGATATCTTGTT
CTTCCGAACTGTTTTAACCCAGTAATCTATGGCATGAGAACCAAGGAAATCAGAGTTCA
AATTGTTAAATATTTGTCTTCTAATAAGGTTAATCTGCATTAA
>YYAB1_or114-1b_allele_A
ATGGATTATAGGACATTCAACTTTTCTCATAACGAATTCTACTTGATTGGATTCACGGAT
CTAAACGAGTTTCGGCAGTATCTGTTCATCCCATTTTTAGTCTTGTTTGTGTTCCAATTG
TTTGCGAATGGAATTATAATGTTTGTGATTATAAAAGAAAGAAAGTTGCATGCTCCCAT
GTACATTCTGATTGGGTTCATTGCAGCTCTGGGCTTTATAGAACCACTATTTCTTCTTCC
AAGAATGTTAGTCAGTTTTCTGTTTGGCAGAAATGTGATTTATAGAAACGAATGTTTCA
TTCAAATGTTTTGTCTGCATATGGCCGGTTTATTCCAGTCAAGTATATTGGTCATGATGG
CAGTGGACCGATTTTTTGCCATCATTTTCCCCTTGCAGTACCATGACTATGTAAACTTTA
AAACCTCATTAATATTCTGTTTGTCTCTTTTTATCCGTAACTTGCTTTATGTTGCCTCAAT
GGTTGGTTTGCTGGGGCCACTATATTTTTGTAAATCAAACGAGATTTACCACTGTGTTTG
TGAGCATACATCTGTTGTTAATTTAGCCTGTGGTGATCTTACTAAGAATTACATGGCTGG
GATAATCGGTTTCAGCTTAACATCAAGCGACTGTGTATTCATTGTCTGTTCTTACATTGT
TATTTTTGTGATTATATTTCGTTCTCCTTCAGGGGAGTCGCGCCAAAAAGCTATCTATAC
ATGTAGCACACATTTAATGGTCTTGGTGGTGGCCTTTCTCTCTGTTGTGGGTGCATTTGC
TGGCTATAGGATTCCTACAATACCTAGAGATCTACGTGTGCTATGTACCTTGGGATATCT
TATTCTTCCGAACTGTTTTAACCCAGTAATCTATGGCATGAGAACCAAGGAAATCAGAG
TTCAAATTGTTAAATATTTATCTTCTAATAAGGTTAATCTACATCAGTGGTCTTCCCTATT
TTTTTCATGAGAGCCACACTGATACAGTAGGTAGAACCCAAAATGAAGAACACTGATC
TACATTAA
>YYAB1_or114-1b_allele_B
ATGGATTATAGGACATTCAACTTTTCTCATAACGAATTCTACTTGATTGGATTCACGGAT
CTAAACGAGTTTCGGCAGTATCTGTTCATCCCATTTTTAGTCTTGTTTGTGTTCCAATTG
TTTGCGAATGGAATTATAATGTTTGTGATTATAAAAGAAAGAAAGTTGCATGCTCCCAT
GTACATTCTGATTGGGTTCATTGCAGCTCTGGGCTTTATAGAACCACTATTTCTTCTTCC

AAAAATGTTAGTCAGTTTTCTGTTTGGCAGAAATGTGATTTATAGAAACGAATGTTTCA
TTCAAATGTTTTGTCTGCATATGGCCGGTTTATTCCAGTCAAGTATATTGGTCATGATGG
CAGTGGACCGATTTTTCGCCATCATTTTCCCCTTGCAGTACCATGACTATGTAAACTTTA
AAACCTCATTAATATTCTGTTTGTCTCTTTTTATCCGTAACTTGCTTTATGTTGCCTCAAT
GGTTGGTTTGCTGGGGCCACTATATTTTTGTAAATCAAACGAGATTTACCACTGTGTTTG
TGAGCATACATCTGTTGTTAATTTAGCCTGTGGTGATCTTACTAAGAATTACATGGCTGG
GATAATCGGTTTCAGCTTAACATCAAGCGACTGTGTATTCATTGTCTGTTCTTACATTGT
TATTTTTGTGATTATATTTCGTTCTCCTTCAGGGGAGTCGCGCCAAAAAGCTATCTATAC
ATGTAGCACACATTTAATGGTCTTGGTTGTGGCCTTTCTCTCTGTTGTAGGTGCATTTGC
TGGCTATAGGATTCCTACAATACCTAGAGATCTACGTGTGCTATGTACCTTGGGATATCT
TATTCTTCCGAACTGTTTTAACCCAGTAATCTATGGCATGAGAACCAAGGAAATCAGAG
TTCAAATTGTTAAATATTTATCTTCTAATAAGGTTAATCTACATCAGTGGTCTTCCCTATT
TTTTTCATGAGAGCCACACTGATACAGTAGGTAGAACCCAAAATGAAGAACACTGATC
TACATTAA
>YYAB2_or114-1a
ATGGATTATAGGACATTAAACTTTTCTCATAACGAATTCTACTTGATTGGATTCACGAGT
CTAAACGAGTTTCGTCAGTATCTGTTCATCCCATTTTTAATCTTGTTTGTGTTCCAATTGT
CTGCGAATGGAATTATAATGTTTGTGGTTATAAAAGAAAGAAAGTTGCATGCTCCCATG
TACATTCTGATTGGGTTCATTGCAGCTCTGGGCTTTATACAACCACTATTTCTTCTTCCA
AGAATGTTAGTCAGTTTTCTCTTTGGCAAAAATATGATTTATAGAAACGAATGTCTCATT
CAAATGTTTTGTATGCATATGGCGGGTTTGTTCCAGTCAAGTATATTGGTCATGATGGCA
GTGGACCGCTTTTTCGCCATCATTTTCCCCTTGCAGTACCATGACTTTGTAACCTTTAAA
ACCTCATTAATATTCTGTTTGTTTCTTTTTATCCGTAACTTGCTTTATGTTACCTCAATGGT
TGGTTTGATTGGGCCACTATATTTTTGTAAATCAAACGAGATTTACCACTGTGTTTGTGA
GCATACATCTGTTGTTAATATAGCCTGTGGTGATCGTACTAAGAATTACATGGCTGGGAT
AATCGGTTTCAGTTTAACAACAAGCGACTGTGTATTCATTTTCTGTTCTTATGTTGTTAT
TTTTGTGATTATGTCTCGTTCTCCTTCAGGGGAGTCGTGGCATAAAGCTATCTATACATG
TAGCACACATTTAATGGCCATGTTTGTGGCCTTTCTCTCTGTTGTGGGTGCATTTGCTGG
CTATAGGATTTCTACAATACCTAAAGATCTACGTGTGATATGTACCTTGGGATATCTTGTT
CTTCCGAACTGTTTTAACCCAGTAATCTATGGCATGAGAACCAAGGAAATCAGAGTTCA
AATTGTTAAATATTTGTCTTCTAATAAGGTTAATCTGCATTAA
>YYAB2_or114-1b
ATGGATTATAGGACATTCAACTTTTCTCATAACGAATTCTACTTGATTGGATTCACGGAT
CTAAACGAGTTTCGGCAGTATCTGTTCATCCCATTTTTAGTCTTGTTTGTGTTCCAATTG
TTTGCGAATGGAATTATAATGTTTGTGATTATAAAAGAAAGAAAGTTGCATGCTCCCAT
GTACATTCTGATTGGGTTCATTGCAGCTCTGGGCTTTATAGAACCACTATTTCTTCTTCC
AAGAATGTTAGTCAGTTTTCTGTTTGGCAGAAATGTGATTTATAGAAACGAATGTTTCA
TTCAAATGTTTTGTCTGCATATGGCCGGTTTATTCCAGTCAAGTATATTGGTCATGATGG
CAGTGGACCGATTTTTTGCCATCATTTTCCCCTTGCAGTACCATGACTATGTAAACTTTA
AAACCTCATTAATATTCTGTTTGTCTCTTTTTATCCGTAACTTGCTTTATGTTGCCTCAAT
GGTTGGTTTGCTGGGGCCACTATATTTTTGTAAATCAAACGAGATTTACCACTGTGTTTG
TGAGCATACATCTGTTGTTAATTTAGCCTGTGGTGATCTTACTAAGAATTACATGGCTGG
GATAATCGGTTTCAGCTTAACATCAAGCGACTGTGTATTCATTGTCTGTTCTTACATTGT
TATTTTTGTGATTATATTTCGTTCTCCTTCAGGGGAGTCGCGCCAAAAAGCTATCTATAC

ATGTAGCACACATTTAATGGTCTTGGTGGTGGCCTTTCTCTCTGTTGTGGGTGCATTTGC
TGGCTATAGGATTCCTACAATACCTAGAGATCTACGTGTGCTATGTACCTTGGGATATCT
TATTCTTCCGAACTGTTTTAACCCAGTAATCTATGGCATGAGAACCAAGGAAATCAGAG
TTCAAATTGTTAAATATTTATCTTCTAATAAGGTTAATCTACATCAGTGGTCTTCCCTATT
TTTTTCATGAGAGCCACACTGATACAGTAGGTAGAACCCAAAATGAAGAACACTGATC
TACATTAA
>ZS2_or114-1a_allele_A
ATGGATTATAGGACATTAAACTTTTCTCATAACGAATTCTACTTGATTGGATTCACGAGT
CTAAACGAGTTTCGTCAGTATCTGTTCATCCCATTTTTAATCTTGTTTGTGTTCCAATTGT
CTGCGAATGGAATTATAATGTTTGTGGTTATAAAAGAAAGAAAGTTGCATGCTCCCATG
TACATTCTGATTGGGTTCATTGCAGCTCTGGGCTTTATACAACCACTATTTCTTCTTCCA
AGAATGTTAGTCAGTTTTCTCTTTGGCAAAAATATGATTTATAGAAACGAATGTCTCATT
CAAATGTTTTGTATGCATATGGCGGGTTTGTTCCAGTCAAGTATATTGGTCATGATGGCA
GTGGACCGCTTTTTCGCCATCATTTTCCCCTTGCAGTACCATGACTTTGTAACCTTTAAA
ACCTCATTAATATTCTGTTTGTTTCTTTTTATCCGTAACTTGCTTTATGTTACCTCAATGGT
TGGTTTGATTGGGCCACTATATTTTTGTAAATCAAACGAGATTTACCACTGTGTTTGTGA
GCATACATCTGTTGTTAATATAGCCTGTGGTGATCGTACTAAGAATTACATGGCTGGGAT
AATCGGTTTCAGTTTAACAACAAGCGACTGTGTATTCATTTTCTGTTCTTATGTTGTTAT
TTTTGTGATTATGTCTCGTTCTCCTTCAGGGGAGTCGTGGCATAAAGCTATCTATACATG
TAGCACACATTTAATGGCCATGTTTGTGGCCTTTCTCTCTGTTGTGGGTGCATTTGCTGG
CTATAGGATTTCTACAATACCTAAAGATCTACGTGTGATATGTACCTTGGGATATCTTGTT
CTTCCGAACTGTTTTAACCCAGTAATCTATGGCATGAGAACCAAGGAAATCAGAGTTCA
AATTGTTAAATATTTGTCTTCTAATAAGGTTAATCTGCATTAA
>ZS2_or114-1a_allele_B
ATGGATTATAGGACATTAAACTTTTCTCATAACGAATTCTACTTGATTGGATTCACGAGT
CTAAACGAGTTTCGTCAGTATCTGTTCATCCCATTTTTAATCTTGTTTGTGTTCCAATTGT
CTGCTAATGGAATTATAATGTTTGTGGTTATAAAAGAAAGAAAGTTGCATGCTCCCATGT
ACATTCTGATTGGGTTCATTGCAGCTCTGGGCTTTATACAACCACTATTTCTTCTTCCAA
GAATGTTAGTCAGTTTTCTCTTTGGCAAAAATATGATTTATAGAAACGAATGTCTCATTC
AAATGTTTTGTATGCATATGGCGGGTTTGTTCCAGTCAAGTATATTGGTCATGATGGCAG
TGGACCGCTTTTTCGCCATCATTTTCCCCTTGCAGTACCATGACTTTGTAACCTTTAAAA
CCTCATTAATATTCTGTTTGTTTCTTTTTATCCGTAACTTGCTTTATGTTACCTCAATGGTT
GGTTTGATTGGGCCACTATATTTTTGTAAATCAAACGAGATTTACCACTGTGTTTGTGAG
CATACATCTGTTGTTAATATAGCCTGTGGTGATCGTACTAAGAATTACATGGCTGGGATA
ATCGGTTTCAGTTTAACAACAAGCGACTGTGTATTCATTTTCTGTTCTTATGTTGTTATTT
TTGTGATTATGTCTCGTTCTCCTTCAGGGGAGTCGTGGCATAAAGCTATCTATACATGTA
GCACACATTTAATGGCCATGTTTGTGGCCTTTCTCTCTGTTGTGGGTGCATTTGCTGGCT
ATAGGATTTCTACAATACCTAAAGATCTACGTGTGATATGTACCTTGGGATATCTTGTTCT
TCCGAACTGTTTTAACCCAGTAATCTATGGCATGAGAACCAAGGAAATCAGAGTTCAA
ATTGTTAAATATTTGTCTTCTAATAAGGTTAATCTGCATTAA
>ZS2_or114-1b_allele_A
ATGGATTATAGGACATTCAACTTTTCTCATAACGAATTCTACTTGATTGGATTCACGGAT
CTAAACGAGTTTCGGCAGTATCTGTTCATCCCATTTTTAGTCTTGTTTGTGTTCCAATTG
TTTGCGAATGGAATTATAATGTTTGTGATTATAAAAGAAAGAAAGTTGCATGCTCCCAT

GTACATTCTGATTGGGTTCATTGCAGCTCTGGGCTTTATAGAACCACTATTTCTTCTTCC
AAGAATGTTAGTCAGTTTTCTGTTTGGCAGAAATGTGATTTATAGAAACGAATGTTTCA
TTCAAATGTTTTGTCTGCATATGGCCGGTTTATTCCAGTCAAGTATATTGGTCATGATGG
CAGTGGACCGATTTTTTGCCATCATTTTCCCCTTGCAGTACCATGACTATGTAAACTTTA
AAACCTCATTAATATTCTGTTTGTCTCTTTTTATCCGTAACTTGCTTTATGTTGCCTCAAT
GGTTGGTTTGCTGGGGCCACTATATTTTTGTAAATCAAACGAGATTTACCACTGTGTTTG
TGAGCATACATCTGTTGTTAATTTAGCCTGTGGTGATCTTACTAAGAATTACATGGCTGG
GATAATCGGTTTCAGCTTAACATCAAGCGACTGTGTATTCATTGTCTGTTCTTACATTGT
AATTTTTGTGATTATATTTCGTTCTCCTTCAGGGGAGTCGCGCCAAAAAGCTATCTATAC
ATGTAGCACACATTTAATGGTCTTGGTGGTGGCCTTTCTCTCTGTTGTGGGTGCATTTGC
TGGCTATAGGATTCCTACAATACCTAGAGATCTACGTGTGCTATGTACCTTGGGATATCT
TATTCTTCCGAACTGTTTTAACCCAGTAATCTATGGCATGAGAACCAAGGAAATCAGAG
TTCAAATTGTTAAATATTTATCTTCTAATAAGGTTAATCTACATCAGTGGTCTTCCCTATT
TTTTTCATGAGAGCCACACTGATACAGTAGGTAGAACCCAAAATGAAGAACACTGATC
TACATTAA
>ZS2_or114-1b_allele_B
ATGGATTATAGGACATTCAACTTTTCTCATAACGAATTCTACTTGATTGGATTCACGGAT
CTAAACGAGTTTCGGCAGTATCTGTTCATCCCATTTTTAATCTTGTTTGTGTTCCAATTG
TTTGCGAATGGAATTATAATGTTTGTGATTATAAAAGAAAGAAAGTTGCATGCTCCCAT
GTACATTCTGATTGGGTTCATTGCAGCTCTGGGCTTTATAGAACCACTATTTCTTCTTCC
AAGAATGTTAGTCAGTTTTCTGTTTGGCAGAAATGTAATTTATAGAAACGAATGTTTCAT
TCAAATGTTTTGTCTGCATATGGCCGGTTTATTCCAGTCAAGTATATTGGTCATGATGGC
AGTGGACCGATTTTTCGCCATCATTTTCCCCTTGCAGTACCATGACTATGTAAACTTTAA
AACCTCATTAATATTCTGTTTGTCTCTTTTTATCCGTAACTTGCTTTATGTTGCCTCAATG
GTTGGTTTGCTGGGGCCACTATATTTTTGTAAATCAAATGAGATTTACCACTGTGTTTGT
GAGCATACATCTGTTGTTAATTTAGCCTGTGGTGATCTTACTAAGAATTACATGGCTGGG
ATAATCGGTTTCAGCTTAACATCAAGCGACTGTGTATTCATTGTCTGTTCTTACATTGTT
ATTTTTGTGATTATATTTCGTTCTCCTTCAGGGGAGTCGCGCCAAAAAGCCATCTATACA
TGTAGCACACATTTAATGGTCTTGGTGGTGGCCTTTCTCTCTGTTGTGGGTGCATTTGCT
GGCTATAGGATTCCTACAATACCTAGAGATCTACGTGTGCTATGTACCTTGGGATATCTT
ATTCTTCCGAACTGTTTTAACCCAGTAATCTATGGCATGAGAACCAAGGAAATCAGAGT
TCAAATTGTTAAATATTTATCTTCTAATAAGGTTAATCTACATCAGTGGTCTTCCCTATTT
TTTTCATGAGAGCCACACTGATACAGTAGGTAGAACCCAAAATGAAGAACACTGATCT
ACATTAA
>DQC1_or114-1
ATGGATTATAGGACATTCAACTTTTCTCATAACGAATTCTACTTGATTGGATTCACGGAT
CTAAAAGAGTTTCGGCAGTATCTGTTCATCCCATTTTTATTCTTGTTTGTGTTCCAATTGT
TTGCGAATGGAATTATAATGTTTGTGATTATAAAAGAAAGAAAGTTGCACGCTCCCATG
TACATTCTGATTGGGTTCATTGCAGCTTTGGGCTTTATAGAACCACTATTTCTTCTTCCA
AGGATGTTAGTCAGTTTTCTGTTTGGCAGAAATATGATTTATAGAAACGAATGTCTCATT
CAAATGTTTTGTCTGCATATGGCTGGTTTGTTCCAGTCAAGTATATTGGTCATGATGGCA
GTGGACCGATTTTTTGCCATCATTTTCCCCTTGCAGTACCATGACTATGTAAACTTTAAA
ACCTCATTAATATTTTGTTTGTCTCTTTATATCCGTAACTTGCTTTATGTTGCCTCAATGGT
TGGTTTGCTGGGGCCACTATATTTTTGTAAATCAAACGAGATTTACCACTGTGTTTGTGA

GCATACATCTGTTGTTAATTTAGCCTGTGGTGATCTTACTAAGAATTACATGGCTGGTGT
AATCGGTTTCAGCTTAACATCAAGCGACTGTGTATTCATTGTCTGTTCTTACATTGTTAT
TTTTGTGATTATATTTCGTTCTCCTTCAGGTGAGTCACGACAAAAAGCTATCTATACATG
TAGCACACATTTAATGGTCTTGGTGGTGGCCTTTCTCTCTGTTGTGGGTGCATTTGCTG
GCTATAGGATTCCTACAATACCTAGAGATCTACGTGTGCTATGTACCTTGGGATATCTTAT
TCTTCCGAACTGTTTTAACCCAGTCATCTATGGCATGAGAACCAAGGAAATCAGAGTTC
AAATTGTTAAATATTTGTCTTCTAATAAGGTTAATCTACATTAA
>DQC2_or114-1_allele_A
ATGGATTATAGGACATTCAACTTTTCTCATAACGAATTCTACTTGATTGGATTCACGGAT
CTAAAAGAGTTTCGGCAGTATCTGTTCATCCCATTTTTATTCTTGTTTGTGTTCCAATTGT
TTGCGAATGGAATTATAATGTTTGTGATTATAAAAGAAAGAAAGTTGCATGCTCCCATG
TACATTCTGATTGGCTTCATTGCAGCTTTGGGCTTTATAGAACCACTATTTCTTCTTCCA
AGGATGTTAGTCAGTTTTCTGTTTGGCAGAAATATGATTTATAGAAACGAATGTCTCATT
CAAATGTTTTGTCTGCATATGGCTGGTTTGTTCCAGTCAAGTATATTGGTCATGATGGCA
GTGGACCGATTTTTCGCCATCATTTTCCCCTTGCAGTACCATGACTATGTAAACTTTAAA
ACCTCATTAATATTCTGTTTGTCTCTTTATATCCGTAACTTGCTTTATGTTGCCTCAATGG
TTGGTTTGCTGGGGCCACTATATTTTTGTAAATCAAACGAGATTTACCACTGTGTTTGTG
AGCATACATCTGTTGTTAATTTAGCCTGTGGTGATCTTACTAAGAATTACATGGCTGGTG
TAATCGGTTTCAGCTTAACATCAAGCGACTGTGTATTCATTGTCTGTTCTTACATTGTTAT
TTTTGTGATTATATTTCGTTCTCCTTCAGGGGAGTCGCGCCAAAAAGCTATCTATACATG
TAGCACACATTTAATGGTCTTGGTGGTGGCCTTTCTCTCTGTTGTGGGTGCATTTGCTG
GCTATAGGATTCCTACAATACCTAGAGATCTACGTGTGCTATGTACCTTGGGATATCTTAT
TCTTCCGAACTGTTTTAACCCAGTAATCTATGGCATGAGAACCAAGGAAATCAGAGTTC
AAATTGTTAAATATTTGTCTTCTAATAAGGTTAATCTACATTAA
>DQC2_or114-1_allele_B
ATGGATTATAGGACATTCAACTTTTCTCATAACGAATTCTACTTGATTGGATTCACGGAT
CTAAAAGAGTTTCGGCAGTATCTGTTCATCCCATTTTTATTCTTGTTTGTGTTCCAATTGT
TTGCGAATGGAATTATAATGTTTGTGATTATAAAAGAAAGAAAGTTGCACGCTCCCATG
TACATTCTGATTGGGTTCATTGCAGCTTTGGGCTTTATAGAACCACTATTTCTTCTTCCA
AGGATGTTAGTCAGTTTTCTGTTTGGCAGAAATATGATTTATAGAAACGAATGTCTCATT
CAAATGTTTTGTCTGCATATGGCTGGTTTGTTCCAGTCAAGTATATTGGTCATGATGGCA
GTGGACCGATTTTTTGCCATCATTTTCCCCTTGCAGTACCATGACTATGTAAACTTTAAA
ACCTCATTAATATTTTGTTTGTCTCTTTATATCCGTAACTTGCTTTATGTTGCCTCAATGGT
TGGTTTGCTGGGGCCACTATATTTTTGTAAATCAAACGAGATTTACCACTGTGTTTGTGA
GCATACATCTGTTGTTAATTTAGCCTGTGGTGATCTTACTAAGAATTACATGGCTGGTGT
AATCGGTTTCAGCTTAACATCAAGCGACTGTGTATTCATTGTCTGTTCTTACATTGTTAT
TTTTGTGATTATATTTCGTTCTCCTTCAGGTGAGTCACGACAAAAAGCTATCTATACATG
TAGCACACATTTAATGGTCTTGGTGGTGGCCTTTCTCTCTGTTGTGGGTGCATTTGCTG
GCTATAGGATTCCTACAATACCTAGAGATCTACGTGTGCTATGTACCTTGGGATATCTTAT
TCTTCCGAACTGTTTTAACCCAGTCATCTATGGCATGAGAACCAAGGAAATCAGAGTTC
AAATTGTTAAATATTTGTCTTCTAATAAGGTTAATCTACATTAA
>HHD1_OR114-1_allele_A
ATGGATTATAGGACATTCAACTTTTCCCATAACGAATTCTACTTGATTGGATTCACGGAT
TTGAAAGAGTTTCGGCAGTATCTGTTCATCCCATTTTTAGTCTTGTTTGTTTTCCAATTG

TTTGCGAATGGAATTATAATGTTTGTGATTATAAAAGAAAGAAAGTTGCATGCTCCCAT
GTACATTCTGATTGGGTTCATTGCGGCTCTGGGCTTTATAGAACCAATATTTCTTCTTCC
AAGAATGTTAGTCAGTTTTCTGTTTGGCAGAAATATGATTTATAGAAACGAATGTCTCAT
TCAAATGTTTTGTCTGCATATGGCGGGTTTGTTCCAGTCAAGTATATTGGTCGGGATGGC
AGTGGACCGATTTTTTGCCATCATTTTCCCCTTGCAGTACCATGAGTATGTAAACTTTAA
AACCTCATTAATATTCTGTTTGTCTCTTTTTATCCGTAACTTGCTTTATGTTGCCTCAATG
GTTGGTTTGCTTGGTCCACTCTATTTTTGTAAATCAAACGAGATTTACCACTGTGTTTGT
GAACACACATCAGTTGTTAATTTAGCCTGTGGTGATCTTACTAAGAATTACGTGGCGGG
TGTAATCGGTTTCAGTTTAACATCAAGCGACTGTGTATTCATTGTCTGTTCTTACATTGT
TATTTTTGTGATTATATTTCGTTCTCCTTCAGGCGAGTCGCGCCAAAAAGCTATCTATAC
ATGTAGCACACATTTAATGGTCTTGGTCGTGGCCTTTCTCTCTGTTGTTGGTGCATTTGC
TGGCTATAGGATTCCTTCAATACCTAGAGATCTACGTGTGCTATGTACCTTGGGATATCTT
ATTCTTCCGAACTGTTTTAACCCAGTAATCTATGGCATGAGAACCAAAGAAATCAGAGT
TCAAATTGTTAAATATTTATCTTCTAATAAGGTTAATCTACATTAA
>HHD1_OR114-1_allele_B
ATGGATTATAGGACATTCAACTTTTCCCATAACGAATTCTACTTGATTGGATTCACGGAT
TTGAAAGAGTTTCGGCAGTATCTGTTCATCCCATTTTTAGTCTTGTTTGTTTTCCAATTG
TTTGCGAATGGAATTATAATGTTTGTGATTATAAAAGAAAGAAAGTTGCATGCTCCCAT
GTACATTCTGATTGGGTTCATTGCGGCTCTGGGCTTTATAGAACCAATATTTCTTCTTCC
AAGAATGTTAGTCAGTTTTCTGTTTAGCAGAAATATGATTTATAGAAACGAATGTCTCAT
TCAAATGTTTTGTCTGCATATGGCGGGTTTGTTCCAGTCAAGTATATTGGTCGGGATGGC
AGTGGACCGATTTTTTGCCATCATTTTCCCCTTGCAGTACCATGAGTATGTAAACTTTAA
AACCTCATTAATATTCTGTTTGTCTCTTTTTATCCGTAACTTGCTTTATGTTGCCTCAATG
GTTGGTTTGCTTGGTCCACTCTATTTTTGTAAATCAAACGAGATTTACCACTGTGTTTGT
GAACACACATCAGTTGTTAATTTAGCCTGTGGTGATCTTACTAAGAATTACGTGGCGGG
TGTAATCGGTTTCAGTTTAACATCAAGCGACTGTGTATTCATTGTCTGTTCTTACATTGT
TATTTTTGTGATTATATTTCGTTCTCCTTCAGGCGAGTCGCGCCAAAAAGCTATCTATAC
ATGTAGCACACATTTAATGGTCTTGGTCGTGGCCTTTCTCTCTGTTGTTGGTGCATTTGC
TGGCTATAGGATTCCTTCAATACCTAGAGATCTACGTGTGCTATGTACCTTGGGATATCTT
ATTCTTCCGAACTGTTTTAACCCAGTAATCTATGGCATGAGAACCAAAGAAATCAGAGT
TCAAATTGTTAAATATTTATCTTCTAATAAGGTTAATCTACATTAA
>HHD5_OR114-1_allele_A
ATGGATTATAGGACATTCAACTTTTCCCATAACGAATTCTACTTGATTGGATTCACGGAT
TTGAAAGAGTTTCGGCAGTATCTGTTCATCCCATTTTTAGTCTTGTTTGTTTTCCAATTG
TTTGCGAATGGAATTATAATGTTTGTGATTATAAAAGAAAGAAAGTTGCATGCTCCCAT
GTACATTCTGATTGGGTTCATTGCGGCTCTGGGCTTTATAGAACCAATATTTCTTCTTCC
AAGAATGTTAGTCAGTTTTCTGTTTGGCAGAAATATGATTTATAGAAACGAATGTCTCAT
TCAAATGTTTTGTCTGCATATGGCGGGTTTGTTCCAGTCAAGTATATTGGTCGGGATGGC
AGTGGACCGATTTTTTGCCATCATTTTCCCCTTGCAGTACCATGAGTATGTAAACTTTAA
AACCTCATTAATATTCTGTTTGTCTCTTTTTATCCGTAACTTGCTTTATGTTGCCTCAATG
GTTGGTTTGCTTGGTCCACTCTATTTTTGTAAATCAAACGAGATTTACCACTGTGTTTGT
GAACACACATCAGTTGTTAATTTAGCCTGTGGTGATCTTACTAAGAATTACGTGGCGGG
TGTAATCGGTTTCAGTTTAACATCAAGCGACTGTGTATTCATTGTCTGTTCTTACATTGT
TATTTTTGTGATTATATTTCGTTCTCCTTCAGGCGAGTCGCGCCAAAAAGCTATCTATAC

ATGTAGCACACATTTAATGGTCTTGGTCGTGGCCTTTCTCTCTGTTGTGGGTGCATTTGC
TGGCTATAGGATTCCTTCAATACCTAGAGATCTACGTGTGCTATGTACCTTGGGATATCTT
ATTCTTCCGAACTGTTTTAACCCAGTAATCTATGGCATGAGAACCAAAGAAATCAGAGT
TCAAATTGTTAAATATTTATCTTCTAATAAGGTTAATCTACATTAA

>HHD5_OR114-1_allele_B

ATGGATTATAGGACATTCAACTTTTCCCATAACGAATTCTACTTGATTGGATTCACGGAT
TTGAAAGAGTTTCGGCAGTATCTGTTCATCCCATTTTTAGTCTTGTTTGTTTTCCAATTG
TTTGCGAATGGAATTATAATGTTTGTGATTATAAAAGAAAGAAAGTTGCATGCTCCCAT
GTACATTCTGATTGGGTTCATTGCGGCTCTGGGCTTTATAGAACCAATATTTCTTCTTCC
AAGAATGTTAGTCAGTTTTCTGTTTGGCAGAAATATGATTTATAGAAACGAATGTCTCAT
TCAAATGTTTTGTCTGCATATGGCGGGTTTGTTCCAGTCAAGTATATTGGTCGGGATGGC
AGTGGACCGATTTTTTGCCATCATTTTCCCCTTGCAGTACCATGAGTATGTAAACTTTAA
AACCTCATTAATATTCTGTTTGTCTCTTTTTATCCGTAACTTGCTTTATGTTGCCTCAATG
GTTGGTTTGCTTGGTCCACTCTATTTTTGTAAATCAAACGAGATTTACCACTGTGTTTGT
GAACACACATCAGTTGTTAATTTAGCCTGTGGTGATCTTACTAAGAATTACGTGGCGGG
TGTAATCGGTTTCAGTTTAACATCAAGCGACTGTGTATTCATTGTCTGTTCTTACATTGT
TATTTTTGTGATTATATTTCGTTCTCCTTCAGGCGAGTCGCGCCAAAAAGCTATCTATAC
ATGTAGCACACATTTAATGGTCTTGGTCGTGGCCTTTCTCTCTGTTGTTGGTGCATTTGC
TGGCTATAGGATTCCTTCAATACCTAGAGATCTACGTGTGCTATGTACCTTGGGATATCTT
ATTCTTCCGAACTGTTTTAACCCAGTAATCTATGGCATGAGAACCAAAGAAATCAGAGT
TCAAATTGTTAAATATTTATCTTCTAATAAGGTTAATCTACATTAA

Additional File 3. Contigs containing or114-1

>utg881_574483_585588
ATTAATGGGCCTCCTCTTCTCATGAAAGCCCAATCACTGATCGCCACATTTTCTTACAC
ACCTGATCGATATTACTCCTGATATCTCTTGCCTGGAGTCGGGCGGAACTATTTCCGCCA
TTTTTCGTTCCGCCTGTGTTCGGTCACTCCGTGACATTAGCTTTCCTGTTTATGCTAGG
GGTGTTAGCTAGGTAGCTGTATTTGGCATATTTATTTAAACCCAATTCTTCTACATCTGT
GTACCTGAGTTATTTTCTGTTTTTCAATAATGTTACTGTTGATACTAGGGTTGTGCCGATA
GACGATAGTATCGTGTATCGACGCTTCAGACATATCGCCAATGGCAGGCTTTCATGGCG
ATAGGTCATGACGATAGTTGGCGAATGGTTATTATTATTATCATCATAGTTTAATATTAAC
ACCCACAATGCATGCTTTTCCTCACATGAGGGTTTGTGGGCTTCACTTTACGCGGAGAG
CTTGTAAAGAGAGAATTCCTTCTTGCACGTACCTGCACTTAATGCGCGTGTCTTGGTCT
CCTTCTACCACTAAATCCAGTGCGCCGCGTCATGAGCAGCTGTGCTTCTCTCTGTCTCA
CGTGCACGCGCTCTCGCATCTGTCCGAAGTCTAAACATAAACTAAAGTAGTAATCCTTA
TGCATTTGTTCAGTTTAATGCGTTTAATGCGAGCTGCGGCAAACTTTACTTTTTGTTTAA
AATATGACACGCTGCATATTAGCGCCTCTCTCTCACTCTCGCGTACGTGCAGAGAGCTG
CGCTCTGTCCGAAGTCAGATCACTAGGTATTAATCCTGTTTATGATATGCATTTCAATGA
GTTGTAGCAACACGTCCCTCGTGTTTAATATATGATTGTATTTAAAATATGATCGCGTTCC
GCTGGACCGATGCGTTTAAAAAGGCACCTCATGAGAGCACCACTGCTTGACACGTGTA
GTCTTCTGCAACAGCACTAAACCAATGCATTGAATTGTTTGTATGTGCGCGCACGTCCG
TGTGCGTGCGCAGATGCATGTTTTTACAGATACTAAGTAATCATGATAGGGTTTTAATGA
CGCGCTCGCATTAATGGCATCAGGTTTAAAAAGGTTGCGGTCATGACGGTGTTGCTCTT
GTTCTTTTTTTGTCCACCCATCAAGTGACTTGTTATAATGAGTAAAAAATTAACAAATAA
AAAAATTTGTAAACTACTGCCCCGCCCCCCGATAATATCGTGTATCGCCGATTTCACAG
GCTGACGATAGGACGATCTGAAAATAGGGAATATCGCCCAACACTAGTTGATACTCTTA
CAAACACAATTATGATGATTACTGATTTTGTCATACGGAATACTCCTGTCAGCAATAACC
TGTTTGTGCTGAATGATGCATCTTATTTGATAACTTTCATTTAGGTGGTCACCGTGCTG
CCAGATATACATAAGTAGGAAAAACATGCAAATTAATTGGCCTTAAAGAACCAAGTTCG
AAAAAATTACTAATACTAACCTACATCTAATCATCACTTTATAGATAGTGATAAGTGTCA
CATTAATTGCTAAAACCATACGTTTAAAGAGGACTATTTTATTTAATAAGAAAAATTTAC
TGAGGTCCGGACCACAGTCACCTCATAGCTGGCTACGGCCATGATGATCACTTTTGCAC
TCCTTCTTCTTCTTCTTCCTTTATTTCTTTACGCCATTTCAGGATCTACGGCTATATTCATG
GCGAGAACAAGTTAATCTATAAACAAGTTTATTCAGACAAGTTGATCCAGACACAGG
TTGGAGGAGGGACAATTTGGCATCTCCAATCTGCATGTTTTTGGCCTGTGGGAGGAAA
CCGGAGTACCCAGAGTAAACCACTTATCCACTCACCTTTAGTAAAGTGTTTATGACAGT
GCCCTGAAGCATGATATTTAATCCAGTATTCCTTAGAGACTGCTCCATCAGTAAAAACT
GTTCATGAACTCCCAAGAATCCTGGAAATCTCTGGAGATATTAACTGTGTAAATAGAAG
GCCTCTCATAACAAGTTGAAAGCTAATTAAATCACCAGATAATGGAAACATCTAGATAA
TTACTAAAATTATGAAGACAGAGTTTTTTTTGAGACACAAATGCCCAGATTTACTAAAC
AGAGCATGTTAGCATGAGAGCACAATTTCAAAAAAGCACAAATGGGAGTGAACGTTTC
TGTGGGTGATTTACTGACAATGTGCACATTAAGGGGGTTTTCACACCTGCCTTGTTTAC
CAGAGTTCGATTGCTCAGTTGGTGCAGTTCGTTTGGGCAGGTGAGAATGCAGCAATCG
AACTCTGGTGCGCACCAAAAGCGGACCAAACAAGCGGACCGAGACCAGCTTGCAGA

GGTGGTCTTGGTACGCTTTCAAACAAACTCTAGAGCGGTTCGTTTGTGGTGAGAACAC
GATCCGAGATCGAACCGACCTAACTGCAAAAGTACTGCGCATTTTGGACTAAACCAGC
TGCTCTAGTCAACTGTGCAGTGCATTATCGGATGAGGAAGTGTTTGTTGACCGTTTTTA
TTAGCAATATTAGCACAATGACAAATCGCGGACATACCTGGAATTGCGAGGAGGTGCG
GGCGGAGCCTTGGAAAATTTTTGTCTTCGCCGTTTGTAGTGCATTAAATCGTTGTTTTC
AAGCCGCATCCACGATTCATTCTGAAAATGAACAGTTTGTCTAGAGTGCTGTATACAAA
CTATGTATAACAACCATGGATACATAATTACAGCGCACAGCCGTCTGTCCATGGTGTCTG
CTGTATTATCCTTCTTTTGTTTACTTCCAGGGGTTCCGTTGGAATTTGCCGCACGTTGAT
TCTGACCAATCGTGAAGCTGTTTAGGAAATACGTTCAAATACATGTGGCCAATAAGTGA
TGTTGATCTTATCACATGACAGAATTTTGGTTCGTTTCAATTGGTGCGGATCAGAGCAAT
CAGTGTGGTGTGAAATGGAACCAAATCTGCTAAAAAAGTAATGTATAATTTTTTGCTTT
TGGTCCGGACCAAATGAACCGAATTACAGATGTGAAAAGCCCTAAAGAACACATCATC
TAATTTCCATCAGAATACTCTCACCTCACAAATTTTGCATCTAAAATGAAACTTACAAGT
TTTATTTAAAGAGACTGTTTATCATTTGCATATGGAAAAAGGTCAGTTGCCAAAATAATT
GGCAAAAATGTCCATGCCGTTTCAGAGCTAATTTTGTGTCATAAGTAAATCCTGACAGT
ATAAATTTATAAACTTTACCAGTATGAAAGAACATCATCACCATTGATCCATAGCAGAGG
ATGGCCTGACCACTTACAGTATTGTGCAAACGTATTTGGCCACCACCAGTTGTTGTTTT
AGCACAATTTTTGCTCTCTTAATTAAGATATGAAAAATTTGGCTTCTTCGGGCAAAAGT
CAGCATTTACTGTGGCCTTAAAGTCCCAGGGGCTTCAGTCCCAGTACTCGCTTTTATTC
AGATTTCACTCAGATTTACATTCATTCAAAACCGGACAATCACACTGCTGTTGCTTTCC
TCGAACATTGCATTTTTGAGATTAAAACATCGATCTTAAAAGTAATAAGTCAGAGTTTAT
GCTGCTTGGCTCAATCCATCAATTACGCAAAGCTGAATCTCCATCACTTAAAGTAAATG
TCTCTGTCCTGGAGTTTCAAACAAAACAAAAGAATCTGGGGATGATATTTGACACTCAT
TTATCATTTGGACCAAAGCTATATTTTAATGTTGCGGAAAAACTGATTAATGCGTTTGTT
TTTTCTTGCATTGATTATTGCTTTGCTCGCTTGAGTTTTCAAAATACCTTTAATACACAAT
ATGTTCAAAACTCAGCTCCCAGAATCCTAACAAGGATGAGAACTAGAGAGCATGTCAC
TCCCATCTTGTAGTCGCTAGATTGGCTTGACGTCAGGTTTCGTATTCATTTGAAAATTCT
TATGCTTACATCTAAAGCCTTAGCCCCACAATATCTATTTGAACTTTTACCAGTTTATACT
CCCAGACGTGAGCTCCGTTCTTTTGAGTTAGGCCTCCTCCTGACTACTCGCATGCGCTC
CATGGGTGACAGGGCTTTCTCTTCCTTTGCTACAAAACCCTGGAACTGATATTAGACAT
GTTAAATCTTTTAGTGCTTTTAAAGCTTCTTTCAAAACCTATATTTTTAAACTGGTTAATA
TGTAATTTTCTCAGATTAGATGTTGTTCTTTTATTTTTCTAGTTATTAATGCATTGTTTACC
TTTTAAAGTTGCACTCTTCTGTGTAAAGCAGTTTGATAATAACCGTTTAAAGGCGCTATA
TAAAATAAAGTTTATAATTTTATATTATTGTTTTTTAGGCTAAGAAGATCCTGCAGTTTTT
TATAGTTCACAAAACAAAAGATTTTATTCAGTCATAATCCTCATCTTCTGTGAGCTGTTA
ACTTATGAGTTCATTACTGCAACACATATTTAATAAAAAAGACAGACTAAAAAGAATGA
TATGCTTACATAATAGCCATTACTCCTTCTCTCACTAACATAATTATACATCTAATTATAGT
ACATCTTTACATTTCCAGAACATTGCCTTTAACTGCCCTCAATGCATACTCATTTGTTAA
AAAAACCCAGTAGAACTCATTGTCCCAACCTTGTATCAGGGGAGGGGAGCAAGGAATA
TTTAAGAGAGGCACCACTTTGTATTAAATCAGTAGGGGAGAGAGATTTTGTAGATGCTG
CTATCGCAGACAAAAACTGTTGTTTTTTATCTTATGTTAAGAATTGCTTTAGTATTTAACT
GATAAAAATATAGATGCTTTATTTATTTCAAATCTGCAAATGAAATGTAATAACATATTTG
ATTTGGACAACATTAATGCTTAAATGCAAGTATATGTTATTTAATGATATATTTTGATAAG
CTAAAGAATTGTTTCTTGTAATAATCTTGTTCTTATGTCTTTACAGTAAACTTTCACCTAA

GGAATGGATTATAGGACATTAAACTTTTCTCATAACGAATTCTACTTGATTGGATTCACG
AGTCTAAACGAGTTTCGTCAGTATCTGTTCATCCCATTTTTAATCTTGTTTGTGTTCCAA
TTGTCTGCGAATGGAATTATAATGTTTGTGATTATAAAAGAAAGAAAGTTGCATGCTCC
CATGTACATTCTGATTGGGTTCATTGCAGCTCTGGGCTTTATACAACCACTATTTCTTCTT
CCAAGAATGTTAGTCAGTTTTCTGTTTGGCAAAAATATGATTTATAGAAACGAATGTCT
CATTCAAATGTTTTGTATGCATATGGCGGGTTTGTTCCAGTCAAGTATATTGGTCATGAT
GGCAGTGGACCGCTTTTTCGCCATCATTTTCCCCTTGCAGTACCATGACTTTGTAACCTT
TAAAACCTCATTAATATTCTGTTTGTTTCTTTTTATCCGTAACTTGCTTTATGTTACCTCA
ATGGTTGGTTTGATTGGGCCACTATATTTTTGTAAATCAAACGAGATTTACCACTGTGTT
TGTGAGCATACATCTGTTGTTAATATAGCCTGTGGTGATCGTACTAAGAATTACATGGCT
GGGATAATCGGTTTCAGTTTAACAACAAGCGACTGTGTATTCATTTTCTGTTCTTATGTT
GTTATTTTTGTGATTATGTCTCGTTCTCCTTCAGGGGAGTCGTGGCATAAAGCTATCTAT
ACATGTAGCACACATTTAATGGCCATGTTTGTGGCCTTTCTCTCTGTTGTGGGTGCATTT
GCTGGCTATAGGATTTCTACAATACCTAAAGATCTACGTGTAATATGTACCTTGGGATATC
TTGTTCTTCCGAACTGTTTTAACCCAGTAATCTATGGCATGAGAACCAAGGAAATCAGA
GTTCAAATTGTTAAATATTTGTCTTCTAATAAGGTTAATCTGCATTAACCCCTATGATATG
GTTTCATTTGTTGTAGGCTATCTTTGTTTTCTGTTCCAACTTACTGCGTACATGGATGATG
TTTGTAATTAGTCTGTAATTTGTACATACATGTTAATAATACATCAACTTTTTGTTAGATT
GATACAGTAGACGAATTGGTAGAACAGATATGCATCTATTCATTCTCTTTTGTGTGTGAT
TGTCATATGCGTACATACCTAAAAGCATCCTGAAATTTTGTTATGACTGTATATGTAACAT
TTAATTCTGGTATGATTATAAACATGCCTTTACAAATAAAATGCAACAAAGTTGTGTTGG
ACTGTTGTGTTTCATTCTGTGAAATGTACTGGCTACTCATGTTTACACTTCATTCATTTTT
TCCAATGACAAACATTTCTTAAGAAATCTGCTATTTGTTGAGGGATTCTGTGTTTTCCTA
GTGTATGAACATGTATTCAGTGACTGCATTTGCCGTTTGCACTTATCAATCTTATTACAAT
TAAATGACTCAATACATTTTGTTTTATGACAGTTTGTGCACTATGCATATTTGCTCATTTA
AAGTACAGTAAGTTTAGATTTAAATATTTCATTATTTCCTTATTTTACAAATGACATACAT
TATATTAAAAGAAATCTGCTTAGTTATTTACTTGCTATTTTTCAAAGTAAACGACACTAC
AGTAAACACATAGTTTTATGTATCGTAGTGCTTCCCCCAGCTTATCTTTCTTATAGAAATG
GTTAAAAAAATTAGCTTGAAGTTCATTATCTGAAAAGTACTCACCTACTTTTTGTTTTAA
CAACCTACAAAGTTTAAAATTCACATGAGGGAGATATACAATTTTCGTGAGGGAGCAG
ACTGTAGGCCATTAACAAGGTACACTCTGTTTTAATTATAAATCCACATTATGTTTTGAA
AGGTAATCTTCTAATATTTTAATTAACCACCAGAGTATACAAACATCTGTGTCCTTCACC
CTGGTGTCAATCAGGATTTTGGAATTTGGCCCCACCTTCTAACCCACGCATGTTTATAGC
ATTTTAAATTTTTTACTGTTACACCAATCCCCACATTTTTAGTGCTTTGTTGTCAGGATCA
AACCCCACCGTGTCAATTCCCCAGCATATGGGTCATATTCTAGCTTCACTTACCTGTGGA
GGATTCCAGAAACAGATGAAGCAATGTCCCCAAGTAAACAGAGCAAGGCACCACTGA
AAACAAATCTGATATTTACAACCAGAAAAAAAATTACACAGAATGTTCATTGCTGCTCTG
AAGTGGAATAAATGCATTCTCACTCATTTACTGCATTTACTCACTTTAATTCACTTTATTT
TCTATTCCTGAGAATAGAAATACGTTGGTAAAAAAAAATTTATTTTGTGTATTTGGTATA
ATACAATGTGTTTGTGTGGTTTATGCTAAAAAAAACACACATTTTTTTCCACATACCGTA
CATTTTTCTAGCTCCAGATATCCTGCATTCCTTAAGAGTTCTGATTTTGTACAAAACTCA
TCGATCTGAAAAACGCTGTTTGCCTGATTGGCCAGCTAAACTGTACATTGTGATTGGCC
TGAATACCTCTGATGTCAGCCGGAAATGTGACGCTTCTTACCAATGTTTGAAAGATTTG
CTCACAATGCAATGCTGACAGGAGTTAGCTTACAGGCTATGAGTCAAAGCTGGAGGAA

TTATGATAATGTTGCTCTTATCCACGTCAATAGGCCCAGGAAGTAAACTGTTGCCTAAA
ATCCAAGAAGTCCAAGAAAAGAGATTTACGTTGGAAACGATAACTCGCATCATCATTTA
CTTTGGGGTTTGTACCTTTTGCATATAGTTGCAAATGTACTGTATGTGCAAAAGAGGCAT
CTGCTGTGCTTTGATGTAGGAAATGTAGGAAAATTGCAGTGGACCTAAAAAGTCTGTA
ATTTGTGCCATCAGCTCTTTGCAGTATAAGCTCCATGCACTTGCACATAATTAGATGTTA
GTGCGATTGAGCGAAAGTCTTTAAAGGCGCTAGATTGAGGTGTTCTAAGTACTGGTATA
ATGGTGGTGTTTTTGCATGCTACATGTCTAAAAACACTTGAAACAATCTGGTACATACA
TAACCTAATTGGGATGCACATTCTTTTAAAACAATTGTTCATAAAACAATGCCATCTGGA
CCGGTAGCCTTATTTGGTTTAATTCTTCTAAGCACTCTGGTTACCTCTCCCTCTTCAATTT
TCATTGCATTAGATCAGGGGTGCTCACACTTTTTTCTTACCAAGGGCCAAAAATGAAAC
CTCACCGAAGGTAGGGGGCTGCAAGTAAATGTTGCTGTGTTATATTAAAATTAAAGTTG
CCCTGATTCTTCTAATTTCAAATATTAAAACAATAAATAAGAAAACATTACTGTGTTTAA
GCATAACTAATGCAGTTTTATTTTCAAAGGTATCTGTTGTAAAAAGAAACAATTTTGCC
AATCATTTTAATTTCCCTTTTGCCTGTGTGCCACAACCTCTCCCTTATTAACCTAATAGTA
ACCGTCTACCCAAGTTCATTGAGTTCATGACGCAAGCTATACACCTTCAAGTATGCATG
TACATTAAAACATTTATTCACTTTAATTCCTTGCATTTAATTTCAATTGACATTTTTGTAAT
GTACAAATAAAACAAAAAATTAAATTTTAGAAAATGTTTAATTAGAAATTGACATCGGC
GTCAATGACGTTTATCCTTTTTCCTTATCTGATGTGGCACGACGGGCCAAATTAAAGATC
ATTGCAGGCCAACTTTGGCCCGCAGGTCCTAGTTTGGGTTCCTCTGCATTAGAGTCTTT
GCAAGTCTCATCATAACTTACATTTTTCACATTATCTATAGCAGGGGTCTCAAACATGGT
GCCCGCTATAGCCAGCACAGAGTCTGTAAGGTGCCCACCAAAGCACATCATATTAATAG
TCTTAATTGAACAAAAGGTTGAAGACGCCTGATCTACAGTATTGTCAATTTTCATTGGG
TTAGAGTCTGTGTTGGTATCATCGTTGCATACAGCATTTGCAATATCTACAGTATTGTCA
AATTGTGAAAAACAGTTTTAAAATTATTAACAAAGGTATGGGGATCCTCGCCAAACCAT
TGTTTCTTTTGTCTGCCAAGCATTGTGTTAAACCCCCTCCATGCACATTCCAAACATATA
TATTATATTCCAAACATATACAGACCCCAGCAAGTACAAAAAACATAATCTGAGAGGGA
ACTAACATTGTATATAAAAAGATAAGTACATTTGGTGTCTGCAAATTTTGGGGGACAAA
ATGTTACAATAATGTAAAAATGAATCAAAAGAGCCCACCCCAATGTCTTTACAATGTTC
TGATGTGGAGATAAAAGGCTTTGTTTATTCCATGCTAGGGTACTGTATTTGGTTGCTAGG
AAGAAAATTTGCATCCACTAGTGTTTATACTCCAAGTCACAAGTAAAACGGTTCAACCC
CCGTGTCTCTACAATGTTCTGATGCAGAGATATAAGGCTGTGTTTATTTGGTTGCTAGGG
TACTGTTTTTGGTTGCTAGGTAAAAAATTGGCATCCACTAGTGATTACACTCCAAGTCA
CGAGTCAAACGGTCCAATCCGTGTCTCTACGATGTTCTGATGCAGAGATATAAGGCTTT
GTTAATTCGGTTGCTAGGGTACTGTATTTGGTTGCTAGGGAAAAAATGGCATCCACTAG
TGATTAGACTCTGATTCACAAGTCAAACGGTCCAACCCCTGTGTCTCTACGATGTTCTG
ATGCAGAGATATAAGGCTTTGTTTATTCTGTTGCTAGGGTATTGTATTTGGTTGCTAGGG
GAGTGGCTTGGGAGTGGCCAACGATGCTGCTCCATTAGCTACTTGCCAGCATGAATGAT
ATAAACCAACCCCCATATCGCCATGACATATAGATTTTAAGATATAACTGTTTGAATTTTT
TTCTAGGCTTCTCAAAAGTGGTTGCTAGGGGCGTGGCTCAATTCCTCTGTAAGGATCAC
GAGAGACTGATTGGATGCCTGAGTACAATGAGCCCACCCCCATGTCTCTATGACAATGT
GGTGCAAAGATATCCAACTGTGAATTTTATGATTGCAGTCTATGGGATATGTTGTGACAC
GAACCCATCCTGTCTAGGGTTGGAAAGGCCAGATGACAAAATATTAAAAGCTAAATTT
CAGTTTTCCACCTCCCGCTCCTGTCCCATTTCACCACAAACACAAGAATAGTTTTAAGA
GTTTTATTCACTCTGTGTGGTCAGAAGCATCTCTTCGGAAAGGGGATAAAGGCCAAAA

TAACAAACATAATAGGAATCTAAATTAATTTAGATTATTAACTTACCTAAGAAAATAAA
TAAAAATACAAAAGAAAAGCACTCTTCTTCCCTCACTCTCTAACTAAAAATAACCATGA
GAAAAGAAGTAAAACAAACTAAAAGGCCATCCCAGACCTACATCTCTGAAAGTAATTA
ATTTAACAAAGGAAACAATAACTGAATGAAATGACACATATGTGCAATTAATAAGAATT
AGGAACGAATAGGAAAGTGTCTTCACAGATTTTACAACAAGAGCCGATCTTAAAGACA
CAATAAAGAGAACAAACTCTTGCATCTACGACCGAATGTGAGCTCCGCAACGCGCTCT
ACCGAAGCTCCTGCACACTAGCCGTTTCTCAATGTCAAGGATACTTCCTTGGCAGGAC
TAGTCCTTACAAGTCACTTCCTTCAGAGGCTAGGCGAGGCTCCTTTTAAGCATTCGGAG
AACACGTTAAATGGAACAGGCTAGCAAGTGCACGTCATTACGTCATTGCGTGATTGAA
AGGTGTGGTTTCGGTGCTGCGCGCATAGGATTGTGGGTGATTTCAGTGCGCGAAGAGC
GCAAAGGATACAAATTTGCATCCTTTCCTGTATATGGGATATTTCTTGAACGAAGGACAT
AGTCCTTGGCTGAAATTTCGAGGATCCTTGACATTGGAACAGTCCTTCGACGGATGTC
GATCACGCAGCATCCTCGAAATTCTAGCTTCCGAGGATCCTTCCTTGACATTGAGAAAT
GGCTACTGTCTTCTCTCGTGCTTTATCCCGTCCTCACTCATTAGTTGCAGCTGCTCCCAA
TCACACGGCACTGCCAGAGATAACGAGAAAA
>utg338_52172_63110_reverse
GAATAAATCTACATAAAAAGTTAAAAACAACATTAAGTGGCTTAAGAACAGCATCACTA
GTGGTCATATAAATGATGTCAGCATAGTCAATAATGGGCAGAATCAATTGAAAAATTAA
CTTCTTTCTGACTTCAAGTGTAAAGCAGTTTACAGATCTATAAAGTGTTTTAAGACAAC
CATATATTCTCTTAGTAATATAGTTAATGTGAGGTTTAAAAGTCAGCTCAGAATCCATCC
ACAGTCCAAGATATTTAAATTTGTCCAATATGACCATAGAAGAACCATCAGCAAAATAC
AGTTGTAAGTTAAAGCACCACTGTGTAGGATCTACTCCCATCTAGCGGTAAAGCTCTAT
ATGACAACCAACTGATTATTAGTTTCTAGCCCCCCCCATTCGGAACGCGTTTTAACTAGT
ACGGTGGCCCTGTCATGCCAAGGTTAACATGGCTTCCAGTAGCTAAAGCAAAAGCAAT
GAAAAAAAATGAACAATTTGCATTGTCCTTTGCCTTTGATCCGTCAAAGCACACAGATT
TATGTTAAACATGTAAAAAGTTTGATTGTCATGATATGTCCGCTTAAAATCACATAAGTT
ACAAAAAGCAATCATAAACGTAGCTTAGACACTCCTTTTTCATCCACGCGAGGAAAAA
TATCACAGGGGCTGTTTTCATCGATCAGATCACAAGTGGACGAGAGAGAGACATTACG
TTTACACTTGGTGTTTAAATCCGTCTCTTTTTGTCCATTTTCGACCGCTTCTCTTCAGATT
ACTGAGGAGATGGTCTGTGGACCAGTTCCTCTCCTGTCATTTACTCATGAGCTGGAGTA
ATGACAGGTTTAAATGGACGCGAAACTAATGTCAGAGTCCAATGCTTATGTTTTAAGTA
AACGTTACATGTCCGTGTATATGTAAGAGCTTTCTCTGATATTGGTGAAATAAGATCGCT
CAACTTTCACACGCTTGTAAAATGAAACGAAAGACGCGCAGATCAGACGCTTTTATCA
ATGAAAGGCTAAAAATAGCGCTGTACACCGTGTGTTTGTGTTAGAGGTCAGAAAAGAT
GAAAAACATTTATCTCAGTACCTCAGATTACATAAATGGGCGGAGAGACGGCGTGTGG
CTGTTCCAAGACATTAAACCACATGCATGTTTACACTAACAAGTGTTACGCATACCCAT
GCTATAGTTAGCCAAGGAACTATAAAACTTCAAGTTTACAACATAAACTGTATAGATGT
AAACGAATATGCTGAATTACCTGTGGTGAAGATAGAAAGTAACTTGAGTACAATGAGC
CCCATCTTGGAAAACTAACTCCAATATTGACTTTGGTCTTGATGTTTTTCCTGTCGCGTT
GTCGTTTAAAATCCCTGTTTCGCTTCCTTGGCGACTTGTTTTAATGTCCTCGAGAATAGT
TCTTCAACAGGCTTTCAAAGCATTAGATCGCAGGTTTATCACGGCGTGCGGCCGAAGG
ATTCAGTCTAACGCAGGTCGCATATCCGGGCTGCATACGTCATCAAGCCTGGTTTATTTA
AATTAACTGAGCATTACATTCGCAAGTCATAAGCATATTACATCAATTTACAATTAACTA
AGAATAAATGTCAGCTTTATAATTGTTAATATTCTGAAATAAGACTGTCTTGATGACGTA

TACCGCCTACACATGCAACCTCCGTAGGCTTCAGCTCGCGGCCAGCTTGAGTGTGCTCT
GAAAGCGCGAAAGGCGGAGCTTGAATTTCAGGAATGTCCCTCGTCGGCGAATGTATTT
CAAAGATGGAGGCACAACATGGATTCAGCCAGAGAGCGCTACGGCGGTATGTATTTTA
AATAGCAGATTCTACACTTACGAGAATACTTTGATTAGTGGGGTGGAAGTAATTACACA
TGAATGAGCACATACTTTTGGAAGAAAACATGGGTTTTTGCTAAGAATTAACTAAAAA
AAGTACACACTGGAGCTTTAAGTGCACATGGTCACAAGTGATGTGATAGATTTACATGT
GATAGATTTGCTGTGATTTCACTCACTGTCTGACAAATGTAAATAAAATCTAAGAGTTC
ATCTGGGTGCACTTGTTATGTACCCCACTAACAGTAGTCTATATATCCATATACATAGTCA
CACAACATAATAACCAGACAAAACATATTTTTATGCAAAATTATTTATTATAAAAAATAT
GCTCAGTTTGTATTTACAAAACCAGAAAACTGGTATTTACTGTGTTTAGCTGTGTTTAC
AGACATATTCAGTGATAGTAATCCCATTTTGTTGTTTATAAAAACTCCTTTCTATTGGAA
ACTGTACTCATTGGTGTACTCAACTGCACTCCAGTTGTATATGGAAGGAGATAATAAGA
GAAATATCAGTATTGAGACTGATAAAATCCTGTTTTAGACAGTAAAACCTGTCACGGGG
CAAGGTTTGTGTTTTGAATGGTTTGGTTTTGTTGGCGTCATGACAACATAATCATCATAT
GTGCAGACTTTAATTAGGAAATATAACTAGTTTTAACAAACATGCCTTACTAAAAACATT
ACTTGTGTGCATTTTGTGGCTAAACAAAGTGCACTGATGTATTTTAAGATATGTTAGTGC
AAGTTGTTTTCCGTTTGGACAGCTCTTACATTTATTTTAGTCTAGGAATAGTCTAATCCC
TGTCCGGGAAACCGCCCCATAGAGTTTATTATTTTCTTTTTTCAGTTTACAGCCATTGCC
ACATTACTCCTCATCAGTTATATTAGAGTCCCATTTCAATGTCACTTTTACCAAATTTAGA
TGATATAAATGACATCATATGCATTCAGTCCTGCGTTTGTTCAATATGTTCAAAACTCAG
CTGCCAGAATCCTAACAAGGATGAGAACTAGAGAGCATGTTATTCCCATCTTGTAGTCC
CTACATTGGCTTCACGTCAGGTTTTGTATTGATTTGAACATTTTTATGCTCACATATAAGG
CCTTATACCTATTGAACTTTTAACAGTTAAGATCCTGCAGTTTTTTATAGTTCACAAAAC
AAAGGATTTTATTCAGTCATAATCCTCATCTTCTGTGAGCTGTTAAAGTGCCCATATTAT
GACTGCTTTTTTCACAAGTTAAATAGGTGTATGAGTTCCATAAAACATGTTTCAAAAGT
TGTTTGCTTGAAATAGCTTGTAGGAAAAGATTGTTACCCATCTCTAGTAGCCTCTGTTTC
AGTGCATTTCAGATTGTGCCGTTTTGAGCTGGTCATTACATATTTATGAGCTGCTGCTCC
TTTGATCACGCCCCACTACTAACATCATGTGCCCGTGCGCATGCTCAGTGGTATCGTGA
GCAGTACAGACCATACTGTATTATTTTTATATATTATTATTAATGGTTTATGTTGCCAACAG
ACAAATCAAGCAAAAAAGTACCACTAATAAGTACACTCCAGGAGAAGAAACTCATGA
CACACAATGATTCCAGAGTACATTATTTTCGCAAATGACAGAAATTTAGACCCGGCGGG
GGATGTATACTGCTTTTGGACCGCGTGCTAATTGCTAGAAGCTAGCGGGAGGTCCGGAC
CTCGGGTGTACGTTAGCCTCTTCAGAAAAGCCGGGTCGGTAAGGCCCGGTCTCGGTTA
GTTTGGCAAAACACTTAGTTTGGCAAAGCACTATTACTTAGTTCATGTAGAATTGTAAA
ATATTTTTTTTTACTTTCGAATCTATTCGAAATTACTATACAATTATAAAGTATAATTTACG
GGATTAGCATCAATGTATGGTCTCTGTTTTGAGATACAGATGCTCTAGCATTGTAAATGT
AGTATTTTGTGACAGATGACAACATGCAAAATATAACGTGACTTCTTACCTTTTGTGTTT
GACATAACGATCGCGAATCGAATCAAGTCTGTTTCAGATGTGTGAATGATCCATGAAAG
TCCAATAAAATCCAATGACCATTTTTGTCCACAGATGCGTATAATCCGTGAAATATAGAT
ACATAGTCTTACATCAGATTTCGCATGAACATCTTATTGTCTACACTCTGATGACTATTTG
CGTCGTAAAACACTGTATATTACTCTTTAGGTTCCAATAAACACGCGTTAAAACTTTGTT
TTTAAAAGAGGGCGGGAGTATAATCCATAATATCCAAATAGCGCTGTTATTTCAGTGAG
ACATGATAACAGCAGGGTATCAGCGAAGTGACTGCTCTGTGCTCTCTCTAGCTCCCTGG
TGCGTGGAGACCTGCGAGCGTGATGGTGGGCGGGAAAATTCAAACGGAATGTGACGA

AACGGCTAGTTACGTCACAACGGAGCTGAGTTTAAACTCGTGCAATCTGAGACTCAAG
GCAGAGGACATTCAGAAACCCGTATCTCACTCAAAACAGCATGGATGGATTTTTTTCCA
AGTTTGTATGCGTGTGGGAGCATCAGAGACACAAATGAACACCCCAAAGTGGGCACTT
TAAGTGATGTTATGAGTTCATTACTGCAACACACATTTAATAAAAAAGACTGATTCAAA
AGAATGATATGCTTACAAAATAGACATTACTCCTTCTACATAATTATATAGTACATCTTTA
CATTTCCAGAATATTGCCTTTGACTGCCCTCAATGCATACTCATTTGTTAAAAAAAAAAC
CCAGTAGAACTCATTGTCCCAACCTTGTATCAGGGGAGGGGAGCAAGGAATATTTAAG
AGAGGCACCACTTTGTATTAAATCAGTAGGGGAGAGAGATTTTGTAGATGCTGCTATTG
CAGACATGCTGTAAAAACGACAAACTGTTGTTTTTATCTTATGTTAAGAATTGCTTTAG
TATTTAACTGGCAAAAATATAGAGGTTTATTTATTTCAAATCTGCACATGAAATGTAATA
AAATATTTAATTTGGACAACATTAATGCTTAAATGCAAGTATATATTATTTAATTATAAATT
TTGATAAACTAAAGAATTGTTTCTTGTAATAATGTCGTTCCTATGCCTTTACAGTGAACT
TTCACCAAAGGAATGGATTATAGGACATTCAACTTTTCTCATAACGAATTCTACTTGATT
GGATTCACGGATCTAAACGAGTTTCGGCAGTATCTGTTCATCCCATTTTTAGTCTTGTTT
GTGTTCCAATTGTTTGCGAATGGAATTATAATGTTTGTGATTATAAAAGAAAGAAAGTT
GCATGCTCCCATGTACATTCTGATTGGGTTCATTGCAGCTCTGGGCTTTATAGAACCACT
ATTTCTTCTTCCAAGAATGTTAGTCAGTTTTCTGTTTGGCAGAAATGTGATTTATAGAAA
CGAATGTTTCATTCAAATGTTTTGTCTGCATATGGCCGGTTTATTCCAGTCAAGTATATTG
GTCATGATGGCAGTGGACCGATTTTTTGCCATCATTTTCCCCTTGCAGTACCATGACTAT
GTAAACTTTAAAACCTCATTAATATTCTGTTTGTCTCTTTTTATCCGTAACTTGCTTTATG
TTGCCTCAATGGTTGGTTTGCTGGGGCCACTATATTTTTGTAAATCAAACGAGATTTACC
ACTGTGTTTGTGAGCATACATCTGTTGTTAATTTAGCCTGTGGTGATCTTACTAAGAATT
ACATGGCTGGGATAATCGGTTTCAGCTTAACATCAAGCGACTGTGTATTCATTGTCTGTT
CTTACATTGTTATTTTTGTGATTATATTTCGTTCTCCTTCAGGGGAGTCGCGCCAAAAAG
CTATCTATACATGTAGCACACATTTAATGGTCTTGGTGGTGGCCTTTCTCTCTGTTGTGG
GTGCATTTGCTGGCTATAGGATTCCTACAATACCTAGAGATCTACGTGTGCTATGTACCT
TGGGATATCTTATTCTTCCGAACTGTTTTAACCCAGTAATCTATGGCATGAGAACCAAGG
AAATCAGAGTTCAAATTGTTAAATATTTATCTTCTAATAAGGTTAATCTACATCAGTGGT
CTTCCCTATTTTTTTCATGAGAGCCACACTGATACAGTAGGTAGAACCCAAAATGAAGA
ACACTGATCTACATTAACCCTGAGATATGGTTTAATTTGTTGTATCTTTGTTCTCTGTTCC
AACTTACTGTGTACATGGATGATGTTTGTAATTAGTCTGTAATTTGTACATACATGTTAAT
AATACATCAACGTTTTTGTTAGATTGATACAGTACATGAATTGGTAGAACATATATGAAT
CTATTCATTCTCTTTTGTGTGTGATTGTCATATGCGTACATAAAAGCAACCTGAAATTTT
GTTATGACTGTATATGTAACATTTAATTCTGGTATGATTATAAACATGCCTTTACAAATAA
AATGCAACAAAGTTGTGTTGGACTGTTGTGTTTATCATTCTGTGAAATGTACTGGCTAC
TCATGTTTACACTTCATTCATTTTTTCCAATGACAAACATTTCTTAAGAAATCTGCTATTT
GTTGAGGGATCCTGTGTTTTCCTAGTGTATGAACATGCATTCAGTGACTGCATTTGCCG
TTTGCACTTATCAATCTTATTACAATTAAATGACTCAATACATTTTGTTTTACGACAGTTT
GTGCACTATGCATATTTGCTCATTTAAAGTACAGTAAGTTTAGATTTAAATATTTCATTAT
TTCATTATTTTACAAATGACATACATTATATTAAAAGAAATCTGCTTAGTTATTTACTTGC
TATTTTTTAAAGTAAACAACCCTACAGTAAACACATAGTTTTATGTATCGTAGTGCTTCC
CCCAGCTTATCTTTCTTATAGAAATGGTTTAAAAATTAAAAATTAGCTTGAAGTTCATTA
TCTGAAAAGTACTCACCTACTTTTTGTTTTAACAACCTACAAAGTTTAAAATTTACATAA
GGGAGATATACAATTTTCGTGAGGGAGCAGACTGTAGGCCATTAACAAGGTACACTTCT

AATAATTTAATTAACCACCAGAGTATACAAACATCTGTGTCCTTCACCCTGGTGTCAATC
TTTCTGGCATTTGGCCCCACCTTCTAACCCAAATTTTTTACTGTTACACCAATTCCCACA
TTTTCAGTGCTTTGTTGTCAGGATCAAACCCCACCGTGTCAATACCCCAGCATATGGGT
CATATTCTAGCTTCATTTACATGTGGAGGATTCCAGAAACAGATGAAGCAATGTCCCCA
AGTAAACAGAGCAAGGCACCACTGAAAACAAATCTGATATTTACAACCAGAAAAAATT
ACACAGAATGTTCATTGCTGCTCTGAAGTGGAATAAATGCATTCTCACTCATTTACTGC
ATTTACTCACTTTAATTCACTTTATTGTCTATTCCTGAGAATAGAAATTCATCTTGCACTG
GCATTACATAACACAATGTAACAAACAATACAAAAGTCCACAACACATCCTTACAAAAT
GAAAACATATACAGCACATTTACAGCACAGTTAAAATAATAGATTATTTAAAGGGCACC
TATTTCATTGGTAAAAAAAACAATGTGTTTGTGTGGTTTATGCTTCAAAAACCACATTAT
TTTCCACATACCATACATTTTTCTAGCTCCAGATATCCTGCATTCCTCAAGAGTTCTGATT
TTGTACAAATCTCATTGATCTGAAAAACGCTGTTTGCCTGATTGGCCAGCTAAACTGTA
CATTGTAACTCTCAATGCAATGCTGACAGGAGTTATAATATATAACATAACATAATATGTC
AGATTACACGTTGCACATAGATGGCTGCACTGTGGTGCCATCTTCCAAGGTAAGGAACT
TAGGTGTGATGTTCGACAGCAACTTATCCTTCGATAGTCATATCGCCAACGTCTGCCGTA
CAGCTTTCTTCCACCTTAGAAATATCTCGAAAATACGCCATATACTGTCTACATCTGACG
CAGAGAAGCTTATCCATGCTTTTATGACCTCTAGAATAGACTATTGTAACTCGCTACTCG
GGGGATGCCATGCAAATCAAGTAAACAAGCTTCAGCTAGTTCAAAACGCTTCCGCAAG
GGTACTTACTCGATCTAAAAAGTACGACCACATAAGCCCAATTCTGGCATCTTTACACT
GGCTACCAGTTAAATATCGCATACAATTTAAAATATCACTACTCACCTACAAAGCTTTAA
ATGGCCTAGCACCCTCATACCTTAGAGAATTACTATCAGAATACAATCCATCACGCACA
CTACGGTCACAAAATTCTGGTCTCTTGATTATCCCTAGACTATCAAAAGTGTCTAAAAG
TGGAAGGTCATTTTCCTACTTAGCCCCTAAGCTCTGGAATGATTTACCAACCGATGTCC
GAGAATCAGACACAGTCGATAATTTTAAATCTAGACTTAAAACTTTTCTCTTCAACAAA
GCATTCGCATAATTTGTCTAGTAAAAGTACTTAACTCGAAATAGTTATTTGTACCGAACA
AAGCACTCGCGGTCATAAAACAGACCAACCAAATAAATAAATAAAAACCTTATCTTAA
CGTATAGTCGGATTGCGATTTTGGAACTTTCGTGTTCTTGTGAATAGTATGCCATACAAA
CCCGTTTGCCACTGAACCTGCATTAACGACGACAGTGAGGCATCCGGCCTTAGTCAAA
CGGGTTGGCACGTACGGTTGGGTTGGGCTTTTGGCGCTTTCTTTATATTGCGAATACTAT
GCCATACAGACCCGTTTGCCACTGAACCTGCATTAACGACGACAGTGGGGCTTCCAGC
CTTAGTCAAACGGGTTGGCACGTATGGTCGGGTTGCGATTTTGGCGCTTTTTTGTGTCT
TGTGAATAGTATGCCATACAGACCCGTTTGCCACTGAACCTGCATTAACGACGACAGTG
GGGCCTCCAGCCTTAGTCAAACGGGTTGGCACGTATGGTCAGGTTGCGATGTTGGCGT
TTTCTCATGATCTTGTAAATAGCATGCAATATAGACCCGTTTGCCACTGAACCTGCATTA
ACGACGACAGTGGGGTTACAGGCCTTAGTCAAACGGGTCTACAGGTTTCATTTTCTCTT
TCTCTTAAAAATCAGAAGGTGACCCTTAGTTACCATATATACCGTCGCAGTGGGCCCCC
AATTTGCAAAATCCTCAACTGTGTATAATATAATTATGCCGCAATAGTTAGTCTGTCTGA
AACTAAGCTGATTAAACCACAACACTGTGTGACACTTGCATTACATGTGAACGGCCCC
TACGCTAATATGATTTTGTTTTTCTCTCCCTGTCCTGTCCTCGACCCTGAGGACAATGGG
ACAAACAGACCCAGTTCCGGTAGATGTGAAAGTCGACTCACCTCTGATCTACTGGTCG
TCCATCACTGTGATGCCCAGCTGATGCCTGACCAACGACCACCGGCAGAACCCGCTTA
AACCCCGCTTAATCCCCGCTTAATCTCCTTATCCGTTTATATGTGTTTATATACATATATAT
CTCCCAAGGGTTTTTCCCTCCTAGGACTTTTTATTTTTTTTTTTCCTCGGCTCAACAACC
CGGGGTTTTTGTTTTTTTTCTCCTAGGGGGTTTTTTACCCCGGGGAGGTAGCCTGCTTG

GGCTTAATTTAGCTTCTTCTCCTAGACGTTACATTAGTAATACGCTCGTTCATAATGTCG
AGTCATAGCCTCAGCAAATTTGACAGCTTATGCTATTGTGTATTATGTTGTGCTATCTGTC
ATTTTTCTGTGCTTTTACTGCTTCTATTAATGTAAAGCTGCTTTGAAACAATTGAGTATTG
TGAAAAGCGCTATATAAATAAAATTGAATTGAATTGAATTGAATTGAATTGAGTTAACTT
ACAGGCTATGAGTCCAAGCAGGAGAAATTATGATAATGTTGGTCTTATCCACGTCAACA
GGCCCAGGAAGTAAACTGTTGCCTAAAATCCAAGAAGTCCAAGAAAAGAGATTTACG
TTGGAAACGATAACTCGCATCATCATTCAATTTGGGGTTTGTACCTTTGGCACATCGTTA
ACACGTACAATGTACTGTATGTGCAATAAAGTCAAAGAGGCATCTGCTGTGCTTTGATT
GAACATGTAGGCAAATTGCAGTGAGTGGATCTAAAAAGTCTGTAATTTGTGCCATCAGC
TCTTTGCAGTATAAGCTCCATGCACTTGCACATATTAGATGTTAGTGCGATTGGGCGAAA
GTCTTTAAAGGCGCTAGATTGAGGTGTTCTAAGTACTGGTATAATGGAGGTGTTTTTGC
ATGCTACATGTCTAAAAACACTTGAAACAATCTGGTACATACATAACCTAATTGGGATG
CACATTCTTTTAAAACAATGCCCTTAACTCTTTCACCGCCAGCATTTTTAAAAAAAAGT
TGCCAGCCAGCGCCAACGTTTTTCATGATTTTCACCAAAGTTTAATGCCTTCCAGAAAA
TGTTCTCCTTCAAATATATAAACATACAATATACCAAATGAAAGAACAGACCCTCTGCTT
TCAAACAAAAAAAACCTTTTCATCCTACCTTGAGTAGTTCTTTTGTAATCAGCTTTTGA
ATATGGGTAGGTTTCTGCAAAAACACCACATTTTGAGCAAAAAGCAGAGATAATTAAAT
TTTTTTGTGACGGACTTTTCATGGAGATCCCATTCAGAGTGATCTTTAAAACAGACATG
GACATGCAGCAACTTGCCAGAGGGCAATACTTCCGGGTTTAAAAAGTTGCGGAAGGG
CGCCACCTAGTGGATAATAGTGGTATTGCGGAAAGACGGAAATACTCGTCATTGGCAG
GAAAGCGTTTTCTCTTGATTGACGAGATATCTTGTCAATGGCGGGGAAAGAGTTAATGC
CATCTGGACCGGTAGCCTTATTTGGTTTAATTCTTCTAAGCACTTTGGTTACCTCTCCCC
CTTCAATTTTCATTGCATTAGATCAGGGCTGCCCACACTTTTTCTTACCAAGGGCCAAA
AATGAAACCTCACTGAAGGTAGTGGGCCGCAAGTAACTGTTGCTGTGTTATATTA
