# Supplementary material for: Species-Specific Duplication and Adaptive Evolution of a Candidate Sex Pheromone Receptor Gene in Weather Loach
Source: Genes (Basel). 2021 Nov 23;12(12):1845. doi: 10.3390/genes12121845 (PMC8701048; doi:10.3390/genes12121845)
Supplement: Supplementary file 1 [file genes-12-01845-s001.zip › Supplementary Materials-Figures and Tables.pdf]

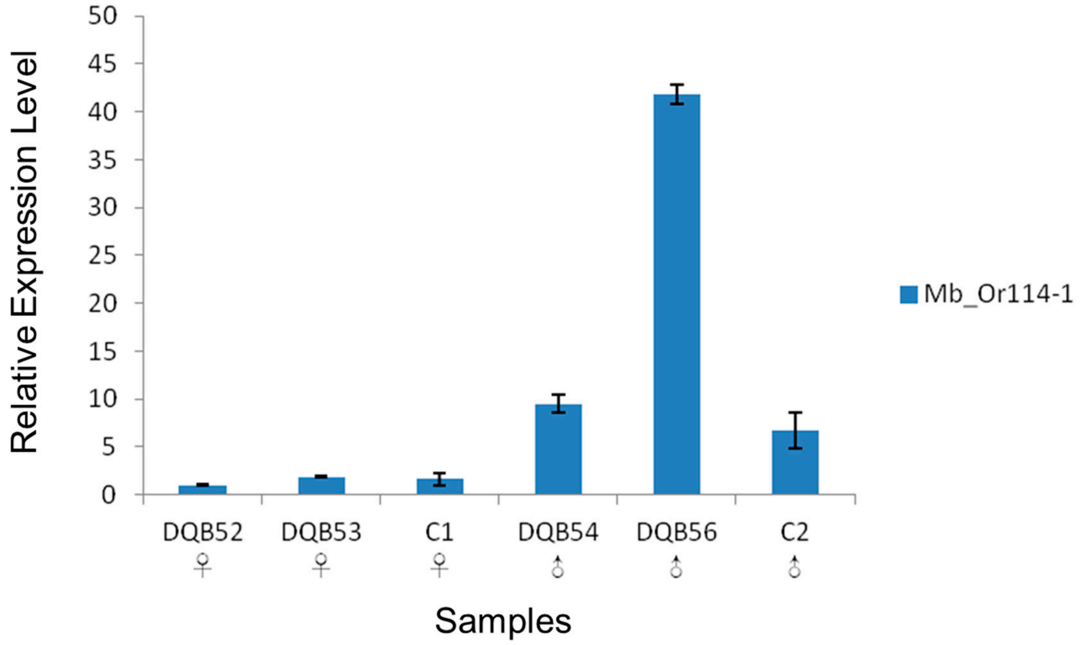

Figure S1. The relative expression levels of *or114-1* in different male and female individuals of *Misgurnus bipartitus*. Based on the relative quantitative results of qRT-PCR ($2^{-\Delta\Delta CT}$). Three technical replicates for each individual, and the error bar is the standard deviation of the three technical replicates. The sample numbers are as Table 1. When comparing expression difference between the male group and the female group, $F = 5.33831E\text{-}4$, $P = 0.00107 < 0.05$; $t = -1.58176$, $P = 0.12719 > 0.05$ (equal variance not assumed), not significant.

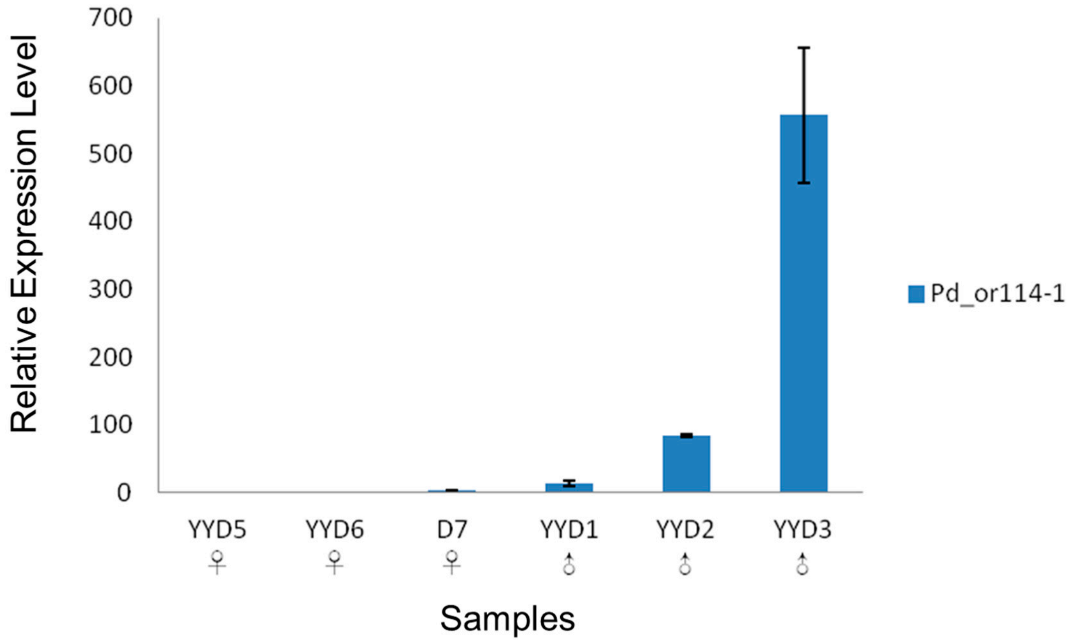

Figure S2. The relative expression levels of *or114-1* in different male and female individuals of *Paramisgurnus dabryanus*. Based on the relative quantitative results of qRT-PCR ($2^{-\triangle\triangle CT}$). Three technical replicates for each individual, and the error bar is the standard deviation of the three technical replicates. The sample numbers are as Table 1. When comparing expression difference between the male group and the female group, F = 1.52706E-5, P = 3.05406E-5 < 0.05; t = -1.27242, P = 0.12719 > 0.05 (equal variance not assumed), not significant.

Table S1. Gene differential expression analysis based on RNA-seq data

Gene	Female-FPKM	Male-FPKM	log2 FC (Male/Female)	*P* value	FDR	Up/Down (Male/Female)
Ma_or114-1a	29.44	289.04	3.3	0	0	Up
Ma_or114-1b	12.42	22.6	0.86	3.97E-09	2.75E-08	/
Mb_or114-1	14.31	30.8	1.11	1.49E-15	3.54E-14	Up
Pd_or114-1	4.57	106.79	4.55	4.70E-259	1.57E-256	Up

NOTE. The RNA-seq data is of the olfactory epithelium of different sex of the three loach species respectively. The differential gene detection is using the PossionDis method. The threshold of up regulation: log2 FC (fold change) ≥ 1 and FDR ≤ 0.001.

Table S2. Test result of RELAX

Model	*log* L	#. params	AIC_c	Branch set	ω_1	ω_2	ω_3
RELAX alternative	-1637.2	25	3325.5	Test	0.00 (85.05%)	0.62 (0.00%)	13.90 (14.95%)
				Reference	0.00 (85.05%)	0.96 (0.00%)	1.24 (14.95%)
RELAX null	-1648.0	24	3345.1	Test	0.00 (82.34%)	1.00 (0.00%)	2.26 (17.66%)
				Reference	0.00 (82.34%)	1.00 (0.00%)	2.26 (17.66%)
Test for selection intensification (K = 12.30) was significant (p = 0.000, LR = 21.62).							

NOTE. The significant result of K>1 indicated that the selection pressure was intensified rather than relaxed in the test branch (*Ma_or114-1a*).

Table S3. Test result of positive selection by aBSREL

a

**Model**	**AIC_C**	**log L**	**Parameters**
Baseline MG94xREV model	3329.85	-1640.43	24
Adaptive aBSREL model	3325.74	-1634.20	28

b

**Name**	**LRT**	**p-value**	**ω distribution over sites**
Ma_or114-1a	6.1500	0.0165	$\omega_1 = 0.00$ (78%)<br>$\omega_2 = 8.84$ (22%)

NOTE. This table lists a statistical summary of the matching data for different models. The Baseline MG94xREV model inferred the likelihood assuming that each branch has the same ω rate type. The Adaptive aBSREL model inferred likelihood under the optimized number of ω rate types for each branch. The likelihood ratio test (LRT) was performed on the likelihood values of the two models, P value = 0.0165 <0.05, the difference was significant. The result indicated that at least one site on the tested branch has undergone positive selection.

Table S4. Test result of positive selection by BUSTED

**Model**	***log* L**	**#. params**	**AIC_c**	**Branch set**	**ω_1**	**ω_2**	**ω_3**
Unconstrained model	-1635.3	29	3330.0	Test	0.00 (29.30%)	0.00 (49.39%)	9.73 (21.31%)
				Background	0.00 (10.44%)	0.00 (85.60%)	3.89 (3.96%)
Constrained model	-1638.4	28	3334.1	Test	0.00 (6.53%)	0.00 (0.00%)	1.00 (93.47%)
				Background	0.00 (10.01%)	0.00 (85.98%)	3.84 (4.02%)
LRT：2△ι=2|-1635.3—1638.4|=6.2，df = 2，p-value = 0.045 < 0.05							

NOTE. This table lists a statistical summary of the matching data for different models. Unconstrained model refers to the alternative model of BUSTED, and Constrained model refers to the null model of BUSTED. The likelihood ratio test (LRT) was performed on the likelihood values of the two models. P value = 0.045 <0.05, the difference was significant. The results indicated that at least one site on the tested branch has undergone positive selection.
